# Use of dexmedetomidine in patients with sepsis: a systematic review and meta-analysis of randomized-controlled trials

**DOI:** 10.1186/s13613-022-01052-2

**Published:** 2022-08-27

**Authors:** Ting Zhang, Qimin Mei, Shabai Dai, Yecheng Liu, Huadong Zhu

**Affiliations:** 1 Emergency Department, State Key Laboratory of Complex Severe and Rare Diseases, Peking Union Medical College Hospital, Chinese Academy of Medical Science and Peking Union Medical College, Beijing, China; 2 Department of Family Medicine & Division of General Internal Medicine, Department of Medicine, State Key Laboratory of Complex Severe and Rare Diseases, Peking Union Medical College Hospital, Chinese Academy of Medical Sciences, Beijing, China; 3 Department of Critical Care Medicine, Peking Union Medical College Hospital, Chinese Academy of Medical Science and Peking Union Medical College, Beijing, China

**Keywords:** Dexmedetomidine, Sepsis, Intensive critical care, Meta-analysis, Mortality, Sedatives, Survival, Inflammatory response

## Abstract

**Background:**

Dexmedetomidine is widely used in patients with sepsis. However, its effect on septic patients remains controversial. The objective of this study was to summarize all randomized controlled trials (RCTs) examining dexmedetomidine use in sepsis patients.

**Methods:**

This systematic review and meta-analysis included RCTs comparing dexmedetomidine with other sedatives in adult sepsis patients. We generated pooled relative risks (RRs) and standardized mean differences and performed trial sequential analysis and a cumulative meta-analysis. The primary outcome was mortality, and the secondary outcomes were the length of the intensive care unit stay, duration of mechanical ventilation, number of ventilation-free days, incidence of total adverse event, incidence of delirium, and levels of interleukin 6, tumor necrosis factor alpha, and alanine aminotransferase.

**Results:**

We included 19 RCTs that enrolled 1929 patients. Compared with other sedatives, dexmedetomidine decreased the all-cause mortality (RR 0.83; 95% confidence interval [CI] [0.69, 0.99]) and inflammatory response (interleukin 6 and tumor necrosis factor alpha levels at 24 h: standardized mean difference (SMD) − 2.15; 95% CI [− 3.25, − 1.05] and SMD − 1.07, 95% CI [− 1.92, − 0.22], respectively). Trial sequential analysis showed that it is not up to required information size. The overall risk adverse events was similar between dexmedetomidine and the other sedatives (RR 1.27, 95% CI [0.69, 2.36]), but dexmedetomidine increased the risk of arrhythmias (RR 1.43, 95% CI [0.59, 3.51]). Length of intensive care unit stay (SMD − 0.22; 95% CI [− 0.85, − 0.41]), duration of mechanical ventilation (SMD 0.12; 95% CI [− 1.10, 1.35]), incidence of delirium (RR 0.98; 95% CI [0.72, 1.33]), and levels of alanine aminotransferase and creatinine at 24 h were not significantly reduced.

**Conclusions:**

Dexmedetomidine in sepsis patients could significantly reduce mortality compared with benzodiazepines but not with propofol. In addition, dexmedetomidine can significantly decrease inflammatory response in patients with sepsis compared with other sedatives. Dexmedetomidine might lead to an increased incidence of arrhythmias, but its safety profile did not show significant differences in the incidence of total adverse events. Future RCTs are needed to determine the sepsis patient population that would benefit most from dexmedetomidine and its optimal dosing regimen.

**Supplementary Information:**

The online version contains supplementary material available at 10.1186/s13613-022-01052-2.

## Background

Sepsis is the systemic inflammatory response syndrome caused by infection. It affects millions of patients per year and has a high risk of mortality, which has become a major global health problem [1] [2] [3]. The Global Burden of Diseases Study showed that sepsis affects at least 49 million patients each year, causing 11 million deaths and accounting for 19.7% deaths worldwide [4] [5]. Epidemiological data showed that over 20% of the septic patients required mechanical ventilation [6], which is associated with enormous costs for health care systems worldwide. The main clinical goal of the 2021 Surviving Sepsis Campaign was to optimize sepsis treatment and improve patient outcomes.

Dexmedetomidine is frequently used for patient comfort and safety, which is an integral component of the therapy concept for mechanically ventilated patients to reduce their anxiety and the stress level associated with tracheal intubation and other invasive interventions [7] [8]. In addition, it can be used to alleviate the symptoms of sepsis-induced encephalopathy in non-ventilated patients [9].

Basic and translational studies showed that among the recommended sedatives, dexmedetomidine (alpha2 receptor agonist) has anti-inflammatory and anti-bacterial effects, which are superior to those of gamma-aminobutyric acid agonists, such as benzodiazepines and propofol [7]. Furthermore, it also reduces neuronal apoptosis and promotes biomimetic sleep—all of which could improve clinical outcomes [10]. For potential risk factors, existing data suggested that a dexmedetomidine loading dose might cause heart arrythmias. However, despite extensive research, the potential benefits and risks of dexmedetomidine in sepsis patients remain controversial.

Recent four meta-analyses have shown controversial results, where two of these studies [11] [12] suggested a positive effect of dexmedetomidine on mortality in sepsis patients, while two other studies [13] [14] did not find a significant difference in mortality between dexmedetomidine and the other sedative agents. However, these conclusions are limited by the number of included studies, and the effects of dexmedetomidine on the incidence of delirium, adverse events, and the length of intensive care unit (ICU) stay remains controversial. Furthermore, trial sequential analysis (TSA) [15] and cumulative meta-analyses were not performed in the previous systematic reviews and meta-analyses.

## Methods

### Protocol and registration

The protocol for this study was pre-registered on PROSPERO (CRD42022303354), and the findings are reported using the Preferred Reporting Items for Systematic Reviews and Meta-Analyses checklist (Additional file 1).

### Systematic search

We conducted a comprehensive search on PubMed, EMBASE, Web of Science, Google Scholar, and unpublished sources including PROSPERO, Clinicaltrials.gov, and the Cochrane Library from inception until February 16, 2022 for randomized controlled trials (RCTs) investigating the role of dexmedetomidine compared with placebo or other sedative agents as therapy in adult sepsis patients. We did not apply language restrictions. We included the following three search terms: “dexmedetomidine,” “sepsis,” and “randomized controlled trials” (Additional file 1: Appendix for the search strategy, appendices S1–S5). We used the Medical Subject Headings database to identify synonyms and examined the reference list of full-text articles for additional relevant studies. We also considered conference proceedings, such as the American Association for the Surgery of Trauma, the Critical Care Medicine, and the European Society of Intensive Care and Emergency Medicine.

### Study selection

Study inclusion criteria are described below. Population: adult patients with sepsis receiving intravenous (IV) sedation in an ICU unit, either with or without mechanical ventilation. Sepsis was defined as per authors’ definition. (Table 1). Intervention: IV dexmedetomidine at any dose. Comparison: received IV sedative drugs regardless of the dose. Outcome: included prespecified outcomes for efficacy on the basis of the meta-analysis group consensus. The primary outcome was all-cause mortality (including ICU, hospital, 7/28/30/90-day mortality). For outcomes reported at multiple timepoints, we chose the longest reported follow-up timepoints. Secondary outcomes included the duration of mechanical ventilation and ventilator-free days; length of ICU stay; biological results (serum interleukin [IL]-6, tumor necrosis factor [TNF]-α, alanine aminotransferase, and creatinine changes at 24 h); incidence of delirium; and incidence of the total adverse events, including tachycardia, bradycardia, and hypotension. Design: RCT.

**Table 1:** Characteristics of included studies

Study author and year	Study design	No. of patients DEX/control	Gender, Male DEX/control	Mean or median age in years DEX/control	Mean or median APACHE II scores DEX/control	Mean or median SOFA scores DEX/control	Sedation goals	Evaluating pain management	Pain management	Ventilation settings	Usage dose in DEX group	Control group	Sepsis case definition	Relevant outcomes collected
Cai et al., 2019	Single site RCT	30/30	20/19	54 ± 17.55/ 58.6 ± 14.95	20.3 ± 4.76/ 21.43 ± 4.52	8.67 ± 1.54/ 8.8 ± 2.36	RASS score of -2 to 0	not reported	not reported	not reported	loading dose of 1 μg/kg, followed by a maintenance dose of 0.2–1 μg/kg/h	Propofol group: loading dose of 1–3 mg/kg, followed by a maintenance dose of 0.05–3 mg/kg/hr	Sepsis-3	Inflammatory cytokine changes; overall mortality on day 28
Chen et al., 2018	Single site RCT	80/80	48/46	47.57 ± 4.48/46.21 ± 4.22	17.74 ± 1.19/ 17.26 ± 1.12	N/A	RASS score of -1 to 0	score of severe patients with pain assessment table was controlled at 0 ~ 1	Remifentanil	not reported	maintenance dose of 0.2–0.7 μg/kg/h	Propofol group: maintenance dose of 0.3–4 mg/kg/hr	Sepsis-2	Inflammatory cytokine changes
Cioccari et al., 2020	Multisite RCT	44/39	29/28	67.7 ± 12.4/ 62.9 ± 16.8	24.9 ± 6.7/ 25.3 ± 7.0	6 (5,10)/ 9 (5,14)	RASS score of -2 to -4	not reported	not reported	not reported	1.12 (0.06–8.0) μg/kg/d; Duration (days): 0.75 (1.7)	Propofol group: 13.56 (4.25–31.7) mg/kg/d; Duration (days): 3.34 (3.27)	Sepsis-2 and septic shock	Vasopressor requirements in the first 48 h; Overall mortality day 28; days of mechanical ventilation; Length of ICU stay; Length of hospital stay; Vasopressor-free at 48 h
Hughes et al., 2021	Multi-center RCT	214/208	121/120	59 (48–68)/ 60 (50–68)	27 (21,32)/ 27 (22,32)	10 (8,13)/ 10 (8,12)	RASS score of -2 to 1	not reported	intermittent opioid boluses or fentanyl infusion	not reported	maintenance dose of 0.2–1.5 μg/kg/h	Propofol group: maintenance dose of 5–50 μg/kg/h	Clinical signs laboratory findings	Overall mortality day 90; Ventilator-free days; days of alive without delirium or coma; Safety end points
Kawazoe et al., 2017	Multisite RCT	100/101	63/64	68(14.9)/ 69(13.6)	23 (18,29)/ 22 (16,29.5)	8 (6,11)/ 9 (5,11)	RASS score of -2 to 0	not reported	DEX group: DEX continuously, and other sedatives control group: propofol, midazolam, and analgesia without DEX	mechanical ventilation for at least 24 h	m [IQR], mg (in the first week): 81 [11, 154.5] ~ 228 [29, 408.5]	Propofol group: m [IQR], mg (in the first week):0 [0, 200] ~ 600 [0, 1077.5]	Sepsis-1	28-day mortality and ventilator-free days; Organ Failure Assessment score (days 1, 2, 4, 6, 8); Sedation control; Occurrence of delirium and coma; Length of ICU stay; Renal function; Inflammation; Nutrition state
Lei et al., 2016	Single site RCT	29/29	17/16	46.5 ± 18.4/ 47.5 ± 15.2	17.9 ± 4.9/ 18.3 ± 4.2	N/A	Ramsay score of 2 to 3	not reported	not reported	not reported	loading dose of 1 μg/kg over 10 min, followed by a maintenance dose of 0.2–0.7 μg/kg/h	Propofol group: loading dose of 1–3 mg/kg over 30–60 s, followed by a maintenance dose of 0.5—4 mg/kg/hr	Sepsis-2	Overall mortality day 28; Length of hospital stays; Changes of myocardial injury markers before and after sedative use
Liu et al., 2020	Single site RCT	100/100	57/58	57 (31–66) / 54 (35–71)	29 (26,37)/ 29 (22,36)	10 (8,13)/ 11 (8,12)	RASS score of -2 to 0	not reported	not reported	not reported	loading dose of 1 μg/kg over 10 min, followed by a maintenance dose of 0.2–0.3 μg/kg/h	Propofol group: loading dose of 1 mg/kg over 10 min, followed by a maintenance dose of 1 to 3 mg/kg/hr	septic shock	Inflammatory cytokine changes; Changes of SCr and BUN; Overall mortality day 28; Length of ICU stays
Memiş et al., 2009	Single site RCT	20/20	14/13	60 (31–80)/ 54 (25–78)	22 ± 5/ 20 ± 8	4.5 ± 2.8/ 4.0 ± 2.9	N/A	not reported	Alfentanil infusion	Ventilator setting: volume or pressure-controlled, no alteration during the study period; only patients with PaO_2_ 80–140 mmHg and PaCO_2_ 35–50 mmHg were included	loading dose of 1 μg/kg over 10 min, followed by a maintenance dose of 0.2–2.5 μg/kg/h	Propofol group: loading dose of 1 mg/kg over 15 min, followed by a maintenance dose of 1 to 3 mg/kg/hr	septic shock	Overall ICU mortality; Length of ICU stay
Meng et al., 2014	Single site RCT	20/20	13/11	56 ± 18/ 51 ± 14	18 ± 4/ 19 ± 4	4.2 ± 1.7/ 4.1 ± 2.4	Ramsay score of 2 to 3	Behavioral Pain Scale (BPS)	Alfentanil: 1.0–3.0 μg/kg/min	not reported	loading dose of 1 μg/kg over 10 min, followed by a maintenance dose of 0.2–2.5 μg/kg/h	Propofol group: loading dose of one mg/kg over 15 min, followed by a maintenance dose of three mg/kg/hr	Sepsis-1	Inflammatory cytokine changes
Pandharipande et al., 2010	Single site RCT	31/32	18/13	60 (46,65)/ 58 (44,66)	30 (26, 34)/ 29 (24, 32)	10 (9,13)/ 9 (8,12)	RASS score of -4 to -2	changes in vital signs, facial expressions, limb movement, ventilator synchrony	Fentanyl, intermittent doses	not reported	maximum 1.5 mcg/kg/hr	Lorazepam group: maximum 10 mg/hr	Sepsis-2	Delirium/coma-free days; Ventilator-free days; Risk of dying at 28 days; Reduced the daily risk of delirium
Sigler et al., 2018	Single site RCT	17/19	13/9	62.5/59	19 (13, 20)/ 16 (12, 19)	11 (7, 14)/ 10 (8, 13)	RASS score of -2 to 2	not reported	Fentanyl infusion, or intermittent opioid boluses	not reported	initiated at 0.2 mcg/kg/hour, titrated every 5 min by 0.1 mcg/kg/hour to a maximum dose of 1.4 mcg/kg/hour	Propofol group: initiated at 5 mcg/kg/minute and titrated every 5 min by 5 mcg/kg/minute	Sepsis-2	Overall mortality day 28; days of mechanical ventilation, Length of ICU stay, Vasopressor infusion
Tasdogan et al., 2009	Single site RCT	20/20	14/11	58 (21–78)/ 50 (19–74)	19 ± 5/ 18 ± 4	4.2 ± 1.8/ 4.0 ± 2.5	N/A	Behavioral Pain Scale (BPS)	Alfentanil, 0.25–1.0 μg/kg/min	Mechanical ventilation setting: DEX/control group Tidal volume (mL/kg): 6.5(6.0–8.6)/6.2(5.8–7.8) Respiratory rate (breaths/ min): 24(19–26)/22(18–26) Fi0_2_(%): 55(40–65)/55(45–70) PEEP (cmH_2_O): 5(5–8)/6 (5–10)	loading dose of 1 μg/kg over 10 min followed by a maintenance dose of 0.2–2.5 μg/kg/h	Propofol group: loading dose of one mg/kg over 15 min, followed by a maintenance dose of one to three mg/kg/hr	Sepsis-1	Biochemical and hemodynamic parameters; Cytokine levels; IAP were recorded before the start of the study and at the 24th and 48th hours
Wang et al., 2016	Single site RCT	28/28	24/24	47.32 ± 14.86/ 51.11 ± 15.15	11.21 ± 3.99/ 11.86 ± 6.87	10.68 ± 5.15/ 11.39 ± 5.19	Ramsay score of 3 to 4	not reported	not reported	not reported	loading dose of 1 μg/kg over 10 min followed by a maintenance dose of 0.2–0.7 μg/kg/h	Propofol group: loading dose of 0.025–1 mg/kg, followed by a maintenance dose of 0.5–4 mg/kg/hr	Sepsis-2	Inflammatory cytokine changes; Overall mortality; Length of ICU stay; Incidence of adverse reactions
Wang et al., 2019	Single site RCT	31/32	17/17	74.13 ± 10.69/	20.97 ± 5.64/20.7 ± 5.85	8.23 ± 1.23/ 8.07 ± 1.46	RASS score of -2 to 0	Critical Care Pain Observation Tool (CPOT), goal: CPOT < 3	Butorphanol tartrate 0.5 ~ 1.0 mg as loading dose, 0.10–0.25 mg/h pumping	not reported	loading dose of 1 μg/kg over 10 min followed by a maintenance dose of 0.2–1 μg/kg/h	Propofol group: loading dose of 1–3 mg/kg over 15 min followed by a maintenance dose of 0.5–4 mg/kg/hr	Sepsis-3	Overall ICU mortality; Length of ICU stay; days of mechanical ventilation
Wei et al., 2020	Single site RCT	60/59	33/30	43.45 ± 7. 86/ 45.21 ± 8. 35	26.43 ± 5.24/ 25.12 ± 5.89	12.37 ± 2.82/ 11.82 ± 2.53	SAS score of 1 to 2	not reported	not reported	not reported	loading dose of 1 μg/kg over 10 min followed by a maintenance dose of 0.2–0.7 μg/kg/h	Propofol group: loading dose of 1–1.5 mg/kg, followed by a maintenance dose of 50–150 μg/kg/hr	Guidelines for the treatment of severe sepsis/ septic shock in China 2014	Overall mortality day 30; Incidence of adverse reactions
Wu et al., 2018	Single site RCT	48/48	30/27	47 ± 10/51 ± 8	21.11 ± 3.73/19.96 ± 4.08	3.15 ± 0.86/ 4.83 ± 1.07	RASS score of -2 to 1	not reported	not reported	Mechanical ventilation setting: SIMV) + PSV, A/C Tidal volume 8–10 mL/kg, Respiratory rate 13–18/min PaCO_2_ 35–50 mmHg, adjust PEEP, FiO_2_ to SpO_2_ of 90%	loading dose of 1 μg/kg over 10 min followed by a maintenance dose of 0.2–0.8 μg/kg/h	Midazolam group: loading dose of 0.1 mg/kg over30 seconds followed by a maintenance dose of 0.03–0.15 mg/kg/hr	Sepsis-3	Inflammatory cytokine changes et al
Zhang et al., 2020	Single site RCT	25/25	NA /NA	59.0 ± 4. 8/ 58.8 ± 4. 8	21 ± 4/ 20 ± 5	8. 8 ± 1. 6/ 8. 6 ± 1. 8	N/A	not reported	not reported	not reported	loading dose of 1 μg/kg over 10 min followed by a maintenance dose of 0.2–0.7 μg/kg/h	Propofol group: loading dose of 0.025–1 mg/kg, followed by a maintenance dose of 0.5–4 mg/kg/hr	Clinical signs: neurological dysfunction	Inflammatory cytokine changes; Overall mortality; days of mechanical ventilation; Incidence of adverse reactions
Zheng et al., 2019	Single site RCT	32/30	18/16	46.05 ± 8.52/45. 76 ± 7. 93	14.25 ± 4.81/14.61 ± 4.35	N/A	N/A	not reported	not reported	Tidal volume was set to 6–8 mL/kg, respiration ratio was set to 1:1–1:1.5, respiration rate was set to 12–18 times/min, inhaled oxygen concentration (FiO2) 50–90%, PEEP 8–10	loading dose of 1 μg/kg over 10 min followed by a maintenance dose of 0.2–0.7 μg/kg/h	Midazolam group: loading dose of 0.05 mg/kg over 10 min, followed by a maintenance dose of 0.03–0.2 mg/kg/hr	Sepsis-3	Inflammatory cytokine changes; Overall mortality day 7; days of mechanical ventilation
Zhou et al., 2017	Single site RCT	40/40	22/23	48.54 ± 4.79/ 48.45 ± 4.82	18.07 ± 4.09/17.89 ± 4.32	N/A	Ramsay score of 2 to 3	not reported	not reported	not reported	loading dose of 1 ug/kg/hr over 10 min followed by a maintenance dose of 0.2–0.7 mg/kg/h	Propofol group: loading dose of 1–3 mg/kg over 30–60 s followed by a maintenance dose of 0.4–5 mg/kg/hr	Sepsis-2	Overall mortality day 28; Length of hospital stay; Changes of myocardial injury markers before and after sedative use

*DEX* dexmedetomidine, *N/A* not applicable, *IAP* intraabdominal pressure, *APACHE II* acute physiology and chronic health evaluation, *SOFA* sequential organ failure assessment score,*FiO*_*2*_ inhaled oxygen concentration, *PaO*_*2*_ patients with arterial oxygen tension, *PaCO*_*2*_ patients with arterial carbon dioxide tension_,_ *PEEP* positive end expiratory pressure, *SpO*_*2*_ oxygen saturation,*Cr* Serum creatinine, *BUN* blood urea nitrogen

^*^Mean (SD)

The exclusion criteria were as follows: (1) conference abstracts, comments, editorials, case reports, and systematic reviews, and articles, where the full text was unavailable; and (2) if two or more studies were based on the same patient cohort, we selected the study with the highest number of patients or the most recently published of the studies.

### Data collection process and data items

Two reviewers (Z and M) aggregated the data independently and in duplicate using a pre-specified standardized data abstraction form. A third reviewer (Liu) adjudicated disagreements. We collected data on trial characteristics, demographic data, acute physiology and chronic health evaluation II (APACHE II) [16], sequential organ failure assessment (SOFA) [3], intervention and control procedures, and outcomes of interest. APACHE II used a point score based on the initial values of 12 routine physiologic measurements, age, and the patient’s previous health status to provide a general measure of disease severity [17].

### Risk of bias assessment in individual studies

We assessed the risk of bias (RoB) independently and in duplicate using the Cochrane Risk of Bias 2.0 tool for RCTs. We used the tool to assess the RoB in the following domains: randomization process, deviations from intended interventions, missing outcome data, measurement of the outcome, and selection of the reported results. We ranked each domain as “low,” “some concerns,” or “high”. We determined the overall RoB for each trial on the basis of the highest risk attributed to any one domain. We assessed the certainty of evidence for each outcome using the Grading of Recommendations, Assessment, Development, and Evaluation (GRADE) approach [18]. In accordance with the GRADE methods, we used terminology consistent with the overall certainty of evidence, which includes stronger language for high certainty of evidence and the less certain language (“probably” or “may”) for moderate or low certainty of evidence. We used the Guideline Development Tool (https://www.gradepro.org) to formulate the summary of findings table.

### Summary measures and synthesis of results

Statistical analyses were performed using Review Manager Software 5 (Review Manager [RevMan] Version 5.4. Copenhagen: The Nordic Cochrane Centre, The Cochrane Collaboration, 2020) and STATA software V.16.0 (STATA Corporation, College Station, TX, USA) [19]. We used DerSimonian and Laird random-effects models to conduct the meta-analysis [20]. We presented the results as the relative risk (RR) for dichotomous outcomes, and we presented the mean difference (MD) or standardized mean difference (SMD) with the 95% confidence interval (CI) to outline continuous outcomes. We also presented the absolute difference with the 95% CI, which we used for the GRADE ratings. The median and interquartile range and the mean and standard deviation were determined in accordance with the methods described by McGrath et al. [21].

We assessed the heterogeneity between the selected trials by visual inspection of the forest plots, the Chi-squared test for homogeneity (where p < 0.1 indicates important heterogeneity), and the I^2^ statistic (for which a value of 50% or greater was considered to reflect potentially important heterogeneity) [22]. Funnel plots were created to assess the publication bias using the Egger’s test. We performed a predefined subgroup analysis comparing studies with a high RoB to those with low RoB as well as comparing the APACHE II scores [17], sedation < 24 h and sedation > 24 h, and control drug (dexmedetomidine vs propofol/others), and another subgroup analysis requested by peer review on the basis of the sedation level [23] [24]. Finally, we conducted a sensitivity analysis to investigate the robustness of the result as requested by peer reviewers, analyzing the subgroup based on the mortality outcome and excluding studies that used benzodiazepines as a comparator.

We conducted a cumulative meta-analysis on the basis of the publication year by updating the pooled risk ratio when the result of a new trial were published for the primary outcome [25]. This statistical method was used to detect the dynamic trend of the association result, and it further supported the meta-analysis conclusion. We conducted a TSA [15] using a random effects model for mortality. For the TSA, we used the statistical significance level of 5%, a power of 80%, and a relative risk reduction of 15%. We used a model variance-based heterogeneity correction, and we performed this analysis using Trial Sequential Analysis v.0.9.5.10 beta software (Copenhagen Trial Unit, Centre for Clinical Intervention Research, Rigshospitalet, Copenhagen, Denmark, https://www.ctu.dk/tsa).

## Results

### Study selection

**Fig. 1:**
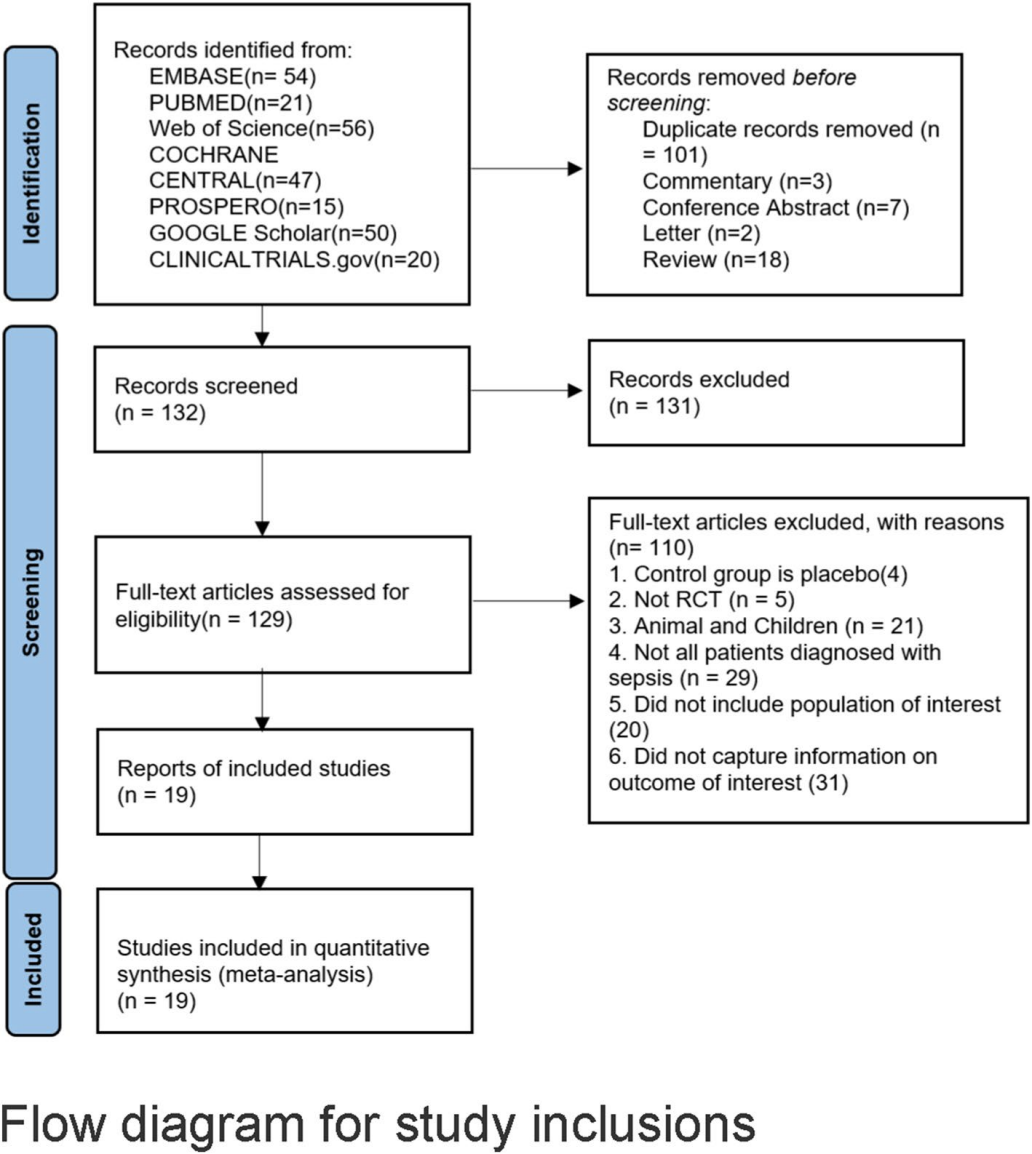
Flow diagram for study inclusions

The searches yielded 263 citations (Fig. 1). After duplicates were removed and the titles and abstracts reviewed, 131 articles were excluded. Among the remaining 132 studies, full-text articles of 129 were available and 110 of them were excluded after reviewing the full-text manuscript. After several review stages, 19 eligible studies were included in the analysis [26–44]. There were 1929 patients included in this study. Baseline characteristics of the included trials are summarized in Table 1.

### Study description

The selected studies were published between 2009 and 2020. The number of included participants from each study ranged from 36 to 422. All patients were in the ICU and met the sepsis criteria. The mean participant age ranged from 43 to 75 years, with male participants accounting for 58.9% of the dexmedetomidine group and 56.8% of the control group. Sepsis was defined as sepsis-1 in three articles [26, 28, 36], sepsis-2 in eight articles [27, 29, 30, 33–35, 41, 44], and sepsis-3 in four articles [37, 38, 40, 43], and as septic shock in two articles [31, 32]. In two articles [39, 42], sepsis was defined in accordance with the 2014 Chinese Guideline of Sepsis and Septic Shock. The dexmedetomidine dose varied among the studies, whereby three [37, 42, 44] out of the six [27, 28, 30, 37, 42, 44] studies administered a loading dose of dexmedetomidine. Sixteen studies used propofol [26, 28–39, 41, 42, 44] and three studies used benzodiazepines as a comparator [27, 40, 43].

**Fig. 2:**
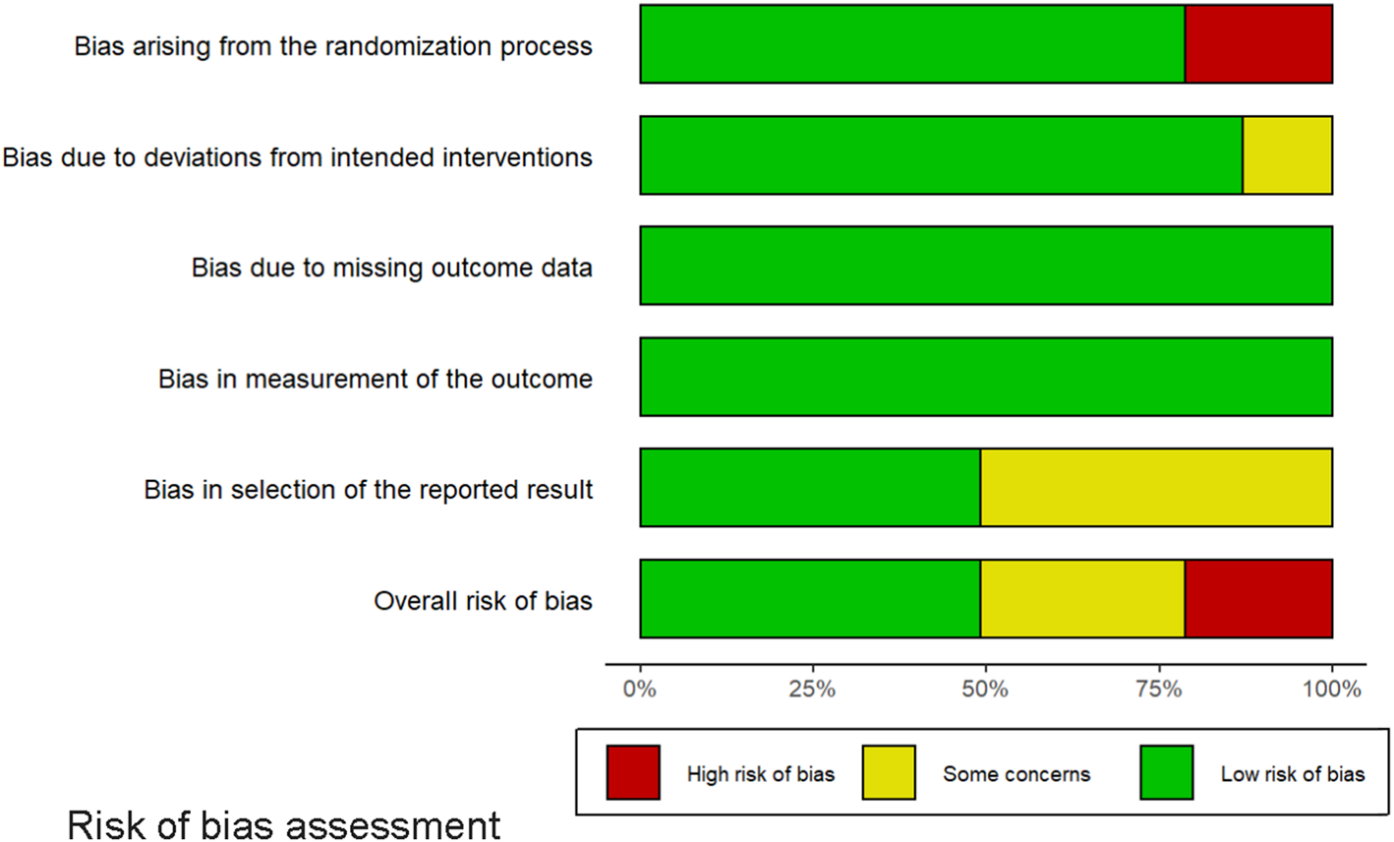
Risk of bias assessment

Five of the included trials had a high RoB [32, 35, 36, 38, 42]. Among them, two studies had a high RoB because of incomplete reporting regarding randomization, intervention descriptions, and reported result selection [32, 42], and three of them had a high RoB due to incomplete reporting of the randomization and concern about selection of the reported results [35, 36, 38]. The other trials had either a low RoB or particular concerns (Fig. 2 and Additional file 1: e-Fig. S1). After discussion among the meta-analysis group, we removed the five studies with a high RoB and then performed the meta-analysis. Table 2 and Additional file 1: e-Tables S2–S7 present the pooled outcomes with the associated GRADE certainty of evidence.

**Table 2:** GRADE summary of findings

Certainty assessment							No of patients		Effect		Certainty	Importance
No of studies	Study design	Risk of bias	Inconsistency	Indirectness	Imprecision	Other considerations	Dexmedetomidine	Other sedatives	Relative (95% CI)	Absolute (95% CI)		
*All-cause mortality at longest follow-up*												
11	Randomised trials	Not serious	Not serious	Not serious	Not serious	None	154/616 (25.0%)	189/606 (31.2%)	RR 0.83 (0.69 to 0.99)	53 fewer per 1,000 (from 97 fewer to 3 fewer)	⨁⨁⨁◯ High	CRITICAL
*Light sedation*												
7	Randomised trials	Not serious	Not serious	Not serious	Not serious	None	130/489 (26.6%)	147/485 (30.3%)	RR 0.90 (0.74 to 1.09)	30 fewer per 1,000 (from 79 fewer to 27 more)	⨁⨁⨁◯ Moderate	CRITICAL
*Deep sedation*												
2	Randomised trials	Not serious	Not serious	Not serious	Seriousa	Sample size	17/75 (22.7%)	26/71 (36.6%)	RR 0.61 (0.30 to 1.23)	143 fewer per 1,000 (from 256 fewer to 84 more)	⨁⨁⨁◯ Moderate	IMPORTANT
*APACHE II ≤20*												
4	Randomised trials	Not serious	Not serious	Not serious	Seriousc	None	18/117 (15.4%)	28/117 (23.9%)	RR 0.67 (0.32 to 1.42)	79 fewer per 1,000 (from 163 fewer to 101 more)	⨁⨁◯◯ Low	IMPORTANT
*APACHE II >20*												
7	Randomised trials	Not serious	Not serious	Not serious	Not serious	None	136/499 (27.3%)	161/489 (32.9%)	RR 0.85 (0.70 to 1.02)	49 fewer per 1,000 (from 99 fewer to 7 more)	⨁⨁⨁⨁ High	CRITICAL
*Dexmedetomidine vs Propofol*												
9	Randomised trials	Not serious	Not serious	Not serious	Not serious	None	145/553 (26.2%)	164/544 (30.1%)	RR 0.89 (0.74 to 1.07)	33 fewer per 1,000 (from 78 fewer to 21 more)	⨁⨁⨁⨁ High	CRITICAL
*Dexmedetomidine vs other sedatives*												
2	Randomised trials	Not serious	Not serious	Not serious	Not serious	Sample size	9/63 (14.3%)	25/62 (40.3%)	RR 0.36 (0.18 to 0.70)	258 fewer per 1,000 (from 331 fewer to 121 fewer)	⨁⨁◯◯ Low -	IMPORTANT

*CI* confidence interval, *RR* risk ratio

a There are differences in the evaluation criteria for sedation

b Wide confidence intervals do not exclude important benefit or harm which lowers our certainty in effect

### Primary outcomes

**Fig. 3:**
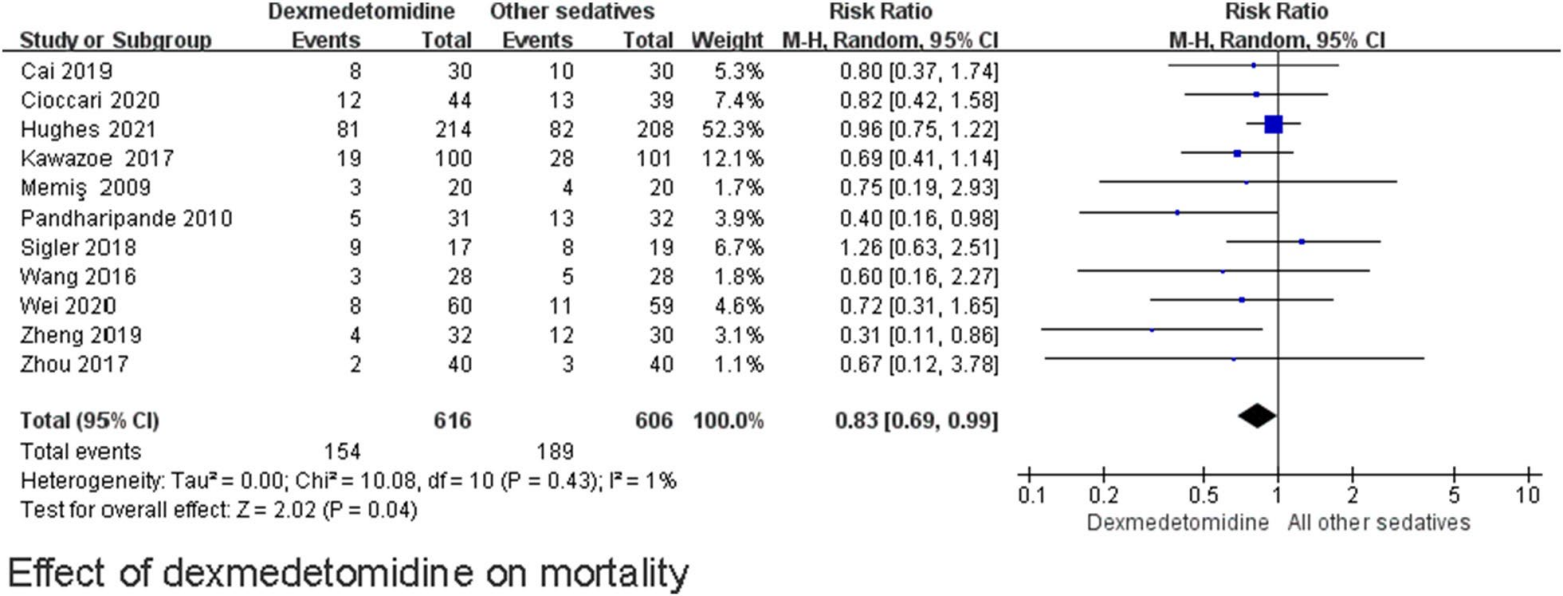
Effect of dexmedetomidine on mortality

**Fig. 4:**
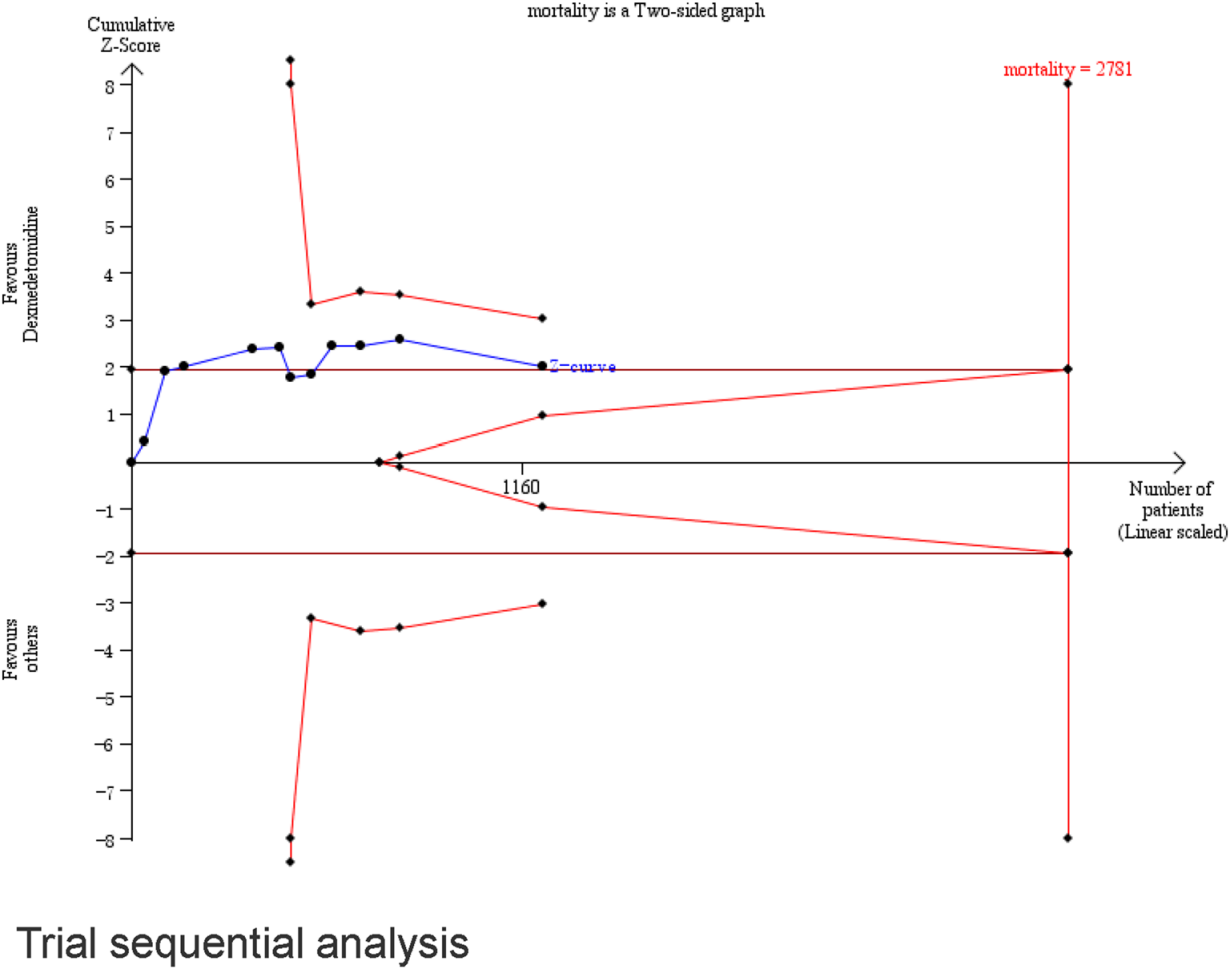
Trial sequential analysis

**Fig. 5:**
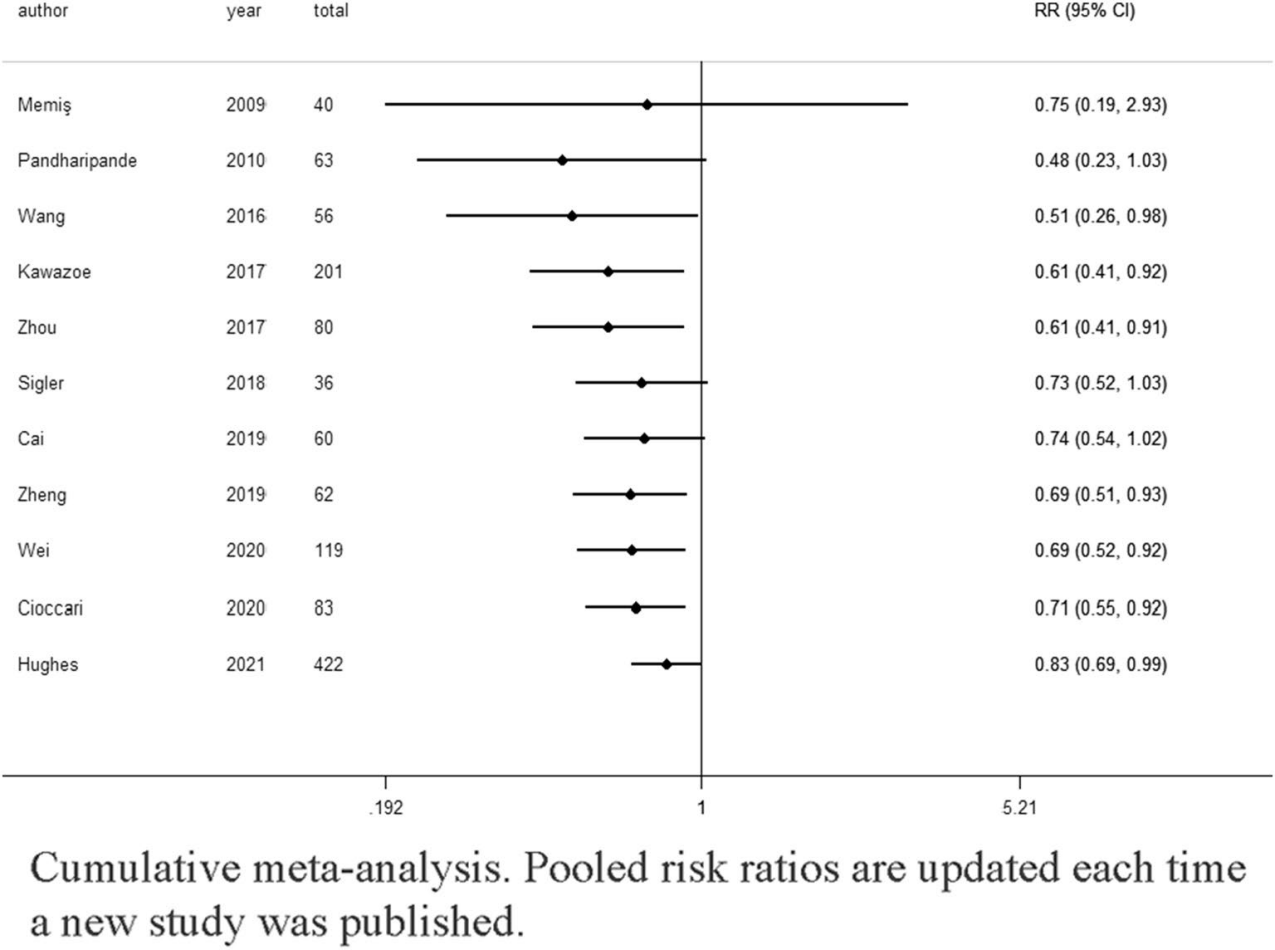
Cumulative meta-analysis. Pooled risk ratios are updated each time a new study was published

Eleven studies (*n* = 1222) showed results for mortality [26–31, 33, 34, 37, 39, 40], among which seven studies explored the 28-day or 30-day mortality, [27–30, 34, 37, 39] two studies focused on the 90-day mortality [26, 33], one study reported the 7-day mortality [40], and one study included ICU mortality of unknown duration [31] (Additional file 1: e-Table S1). A pooled analysis showed that the dexmedetomidine group had a lower occurrence of mortality (RR 0.83; 95% CI [0.69, 0.99]; high certainty) compared with the control group, with no significant heterogeneity (I^2^ = 1%) (Fig. 3). Table 2 shows the summary of findings for all outcomes including the certainty of evidence. Using a funnel plot and Egger’s test (Additional file 1: e-Fig. S2), we did not find any publication bias. The TSA results demonstrated that the information size needed to detect an intervention effect was 2781 patients. The cumulative Z curve did not cross either the conventional boundary for benefit or the trial sequential monitoring boundary for benefit (Fig. 4). A cumulative meta-analysis was conducted to assess changes over time (Fig. 5). A statistically significant decrease in mortality was first observed in studies that were performed from 2009 to 2016 (RR 0.51 95% CI [0.26, 0.98]). As the number of studies increased, the RR value approached 1.

A subgroup analysis was conducted on the basis of APACHE II scores ≤ 20 and > 20, control drug (dexmedetomidine vs other sedatives), and sedation level (deep or light). We found that the patients’ APACHE II scores in each study (≤ 20 or > 20) had no significant effect on mortality (Additional file 1: e-Fig. S3a). In addition, the sedation level (deep or light) did not demonstrate any credible subgroup effects (Additional file 1: e-Fig. S3b). Dexmedetomidine significantly reduced sepsis patients mortality compared with benzodiazepines but not with propofol (RR 0.36, 95% CI [0.18, 0.70]) except for propofol (RR 0.89, 95% CI [0.74, 1.07]; Additional file 1: e-Fig. S3c).

The sensitivity analyses excluding the study reporting 7-day mortality[40] showed that there was no significant difference between the dexmedetomidine and the other sedatives on mortality (RR 0.86; 95% CI [0.72, 1.03]; Additional file 1: e-Fig. S4a). The sensitivity analyses excluding the study reporting 90-day mortality[33] showed that the use of dexmedetomidine was associated with lower mortality compared to other sedatives (RR 0.71; 95% CI [0.55, 0.92], Additional file 1: e-Fig. S4b). After excluding the two studies reporting 7-day mortality[40] and 90-day mortality[33], the use of dexmedetomidine was also associated with lower mortality (RR 0.75; 95% CI [0.58, 0.98], Additional file 1: e-Fig. S4c).

### Secondary outcomes

Length of ICU stay.

Nine studies (n = 659) [26–31, 34, 37, 45] included the length of ICU stay in their evaluation index. Our results indicated that dexmedetomidine did not reduce the length of the ICU stay compared with the other sedatives (SMD − 0.22; 95% CI [− 0.85, 0.41], high certainty) (Additional file 1: e-Fig.SS5a). We performed sensitivity analyses excluding Pandharipande’s study [27] that compared dexmedetomidine to benzodiazepines, and we found no substantially altered pooled estimates or conclusions (SMD − 0.23; 95% CI [− 0.87, 0.40], Additional file 1: e-Fig. S5b).

Duration of mechanical ventilation.

Six studies (n = 460) [26, 28, 30, 34, 36, 37] explored the impact of dexmedetomidine on the duration of mechanical ventilation. The meta-analysis did not show a reduction in mechanical ventilation time with dexmedetomidine use compared with that with the use of other sedatives (SMD 0.12; 95% CI [− 1.10, 1.35], high certainty) (Additional file 1: e-Fig. S6).

Duration of ventilator-free days.

Three studies (*n* = 686) [27, 28, 33] included ventilator-free days as indicator, and the meta-analysis results indicated that dexmedetomidine did not increase ventilator-free days compared with the other sedatives (MD 1.68; 95% CI [− 1.50, 4.85], very low certainty) (Additional file 1: e-Fig. S7a). After excluding Pandharipande’s study [27], a sensitivity analysis was conducted, and the results did not change significantly (SMD 0.29; 95% CI [− 1.81, 2.39]; Additional file 1: e-Fig. S7b).

IL-6, TNF-α, alanine aminotransferase, and creatinine level changes at 24 h.

Four studies (*n* = 352) reported the 24-h changes in IL-6 and TNF-α levels [26, 41, 43, 44]. Three studies (*n* = 219) reported the 24-h changes in alanine aminotransferase, and creatinine levels [31, 37, 39]. Random-effect models were used in the four outcomes, and the results showed significantly lower IL-6 and TNF-α levels at 24 h in the dexmedetomidine group compared with those in the other sedatives group (SMD − 2.15; 95% CI [− 3.25, − 1.05], low certainty; SMD − 1.07; 95% CI [− 1.92, − 0.22], moderate certainty; Additional file 1: e-Figs. S8a and S9a). However, random model analysis indicated that dexmedetomidine did not lead to a significant change in alanine aminotransferase and creatinine levels at 24 h (*p* = 0.17 and 0.30, respectively; low certainty; e-Fig. S10). The sensitivity analysis excluded Wu’s study [43] used benzodiazepines as a comparator and the results did not change (IL-6: SMD − 2.50; 95% CI [− 4.11, − 0.90]; TNF-α: SMD − 0.58; 95% CI [− 0.83, − 0.32], e-Figs. S8b and S9b).

### Incidence of delirium

Two studies (*n* = 264) [28, 37] explored the incidence of delirium related to dexmedetomidine. Overall, 45/131 (34.35%) patients in the dexmedetomidine group reported that they experienced delirium compared with 46/133 (34.59%) patients in the control group. The meta-analysis showed that dexmedetomidine was not significantly associated with a lower risk of delirium compared with the other sedation types (risk ratio 0.98; 95% CI [0.72, 1.33], low certainty; Additional file 1: e-Fig. S11).

### Overall incidence of adverse events

Six studies (n = 581) included the incidence of adverse events [27, 28, 30, 37, 39, 44]. There was no difference in the incidence of adverse events between the dexmedetomidine and propofol groups (RR 1.27, 95% CI [0.69, 2.36], moderate certainty; Additional file 1: e-Fig. S12a). We performed sensitivity analyses excluding Pandharipande’s study [27] and found no substantial changed in the pooled estimates (RR 1.43, 95% CI [0.59, 3.51], Additional file 1: e-Fig. S12b). For arrhythmia and hypotension, the pooled RRs were 2.69 (95%CI [1.19, 6.08], high certainty; e-Fig. S12c) and 1.04 (95% CI [0.46, 2.36], low certainty; Additional file 1: e-Fig. S12d). The research findings showed that dexmedetomidine was significantly associated with a higher risk of arrhythmia but not with a higher risk of hypotension compared with other sedatives.

## Discussion

This systematic review and meta-analysis showed that dexmedetomidine sedation in sepsis patients could significantly decrease mortality and IL-6 and TNF-α levels at 24 h compared with other sedatives. Dexmedetomidine might lead to an increased incidence of arrythmias, but it was not associated with an increased incidence of total adverse events. There were no significant differences in the length of ICU stay, duration of mechanical ventilation, incidence of delirium, and the alanine aminotransferase or creatinine at 24 h. Considering the differences in pharmacological profiles, dexmedetomidine has known strengths including its anesthesia-inducing effect without inhibiting respiration, its anti-inflammation effects, and its low allergenic potential compared with propofol [46]. Dexmedetomidine already has a wide indication field in clinical practice, while propofol was not as widely used in septic shock patients [47, 48]. This study demonstrated that dexmedetomidine has advantages in treating sepsis patients by improving their overall survival.

Several systematic reviews and meta-analyses on this research topic have been previously conducted [11] [12] [13] [49] [14]. Among previous meta-analyses, Huang et al. was the most comprehensive study [13], and it included 15 RCTs with 1,871 patients in the analysis. Huang et al. showed that dexmedetomidine use did not significantly reduce mortality (RR 0.97, 95%CI [0.83, 1.13]) [13]. In Huang et al.’s study, nearly half of the studies were assessed as having a high RoB using the Cochrane Risk of Bias 2.0 tool, and we suspect that this non-significant result may be influenced by these high-RoB studies. A strength of our meta-analysis is that we systematically reviewed the current literature on the basis of previous meta-analyses and excluded studies with a high RoB. Our cumulative meta-analysis for the primary outcomes showed that from a dynamic perspective, although the RR value changed over time, the conclusion was relatively stable over time, and an advantage of dexmedetomidine use in treating sepsis patients was observed.

Comparing the safety profile of dexmedetomidine with that of the other sedation types, there were no significant differences in the incidence of the total adverse events in sepsis patients, although the incidence of arrhythmia was significantly increased. This finding was not reported in previous studies. Theoretically, dexmedetomidine is an alpha2-adrenoceptor agonist that causes vasodilation and decreases the sympathetic response [50] and, therefore, potentially induces hemodynamic side effects. A possible explanation for our research findings is that only three [37, 42, 44] out of the six [27, 28, 30, 37, 42, 44] studies administered a loading dose of dexmedetomidine, which is associated with higher risk of arrhythmia due to a decrease in cardiac output that occurred following the loading dose secondary to a transient afterload increase caused by alpha2-adrenoceptor-mediated vasoconstriction [51]. The incidence of arrhythmia may be reduced by eliminating a dexmedetomidine loading dose, and close hemodynamic monitoring is still recommended.

A large amount of evidence has demonstrated the stimulating effect of dexmedetomidine on the central and peripheral receptors, causing a reduction in sympathetic nerve activity and plasma catecholamine concentration [52]. Its ability to reduce sympathetic tone and indirectly increase the parasympathetic activity is important in inhibiting inflammatory factor release and reducing cell apoptosis, thereby reducing the occurrence of inflammation and sepsis [53]. Results of our meta-analysis also suggest that 24 h after receiving dexmedetomidine, patients’ TNF-α and IL-6 levels were significantly lower compared with those of the control group. However, our meta-analysis results were not consistent with those of previous reports [54, 55], which showed that dexmedetomidine prevents liver and kidney damage resulting from sepsis. Further research is needed to confirm these results. In addition, the sample size included in this study was small.

This systematic review and meta-analysis have several strengths including a protocol that was written a priori, a comprehensive literature search including unpublished sources, independent screening, and data abstractions, and use of the GRADE assessment of the certainty of evidence.

However, there are also some limitations to this study. First, there was a lack of individual patient data, and we were unable to conduct the pre-planned subgroup analyses using the patient baseline characteristics, such as the underlying etiology of sepsis. Because there was a partial lack study data, we had to change the protocol regarding ventilator free-days as a co-primary outcome, and we could not conduct the pre-planned subgroup analyses on the basis of sedation < 24 h and sedation > 24 h. In addition, only a small number of studies reported data on pain management (8) and ventilation settings (5), and we were unable to complete the subgroup analysis on these items. Second, the variations in sepsis definition, dexmedetomidine regimens, sedation levels, sedation substances used as a comparator, adjunctive therapies (e.g., pain management and ventilation settings), and mortality timeline among the included studies might have caused the clinical heterogeneity, although the levels of statistical heterogeneity were low across all studies. Furthermore, the required sample size was not attained (1222 patients were in the analysis but 2781 patients were needed), although recent studies had a major impact on the CI ranges.

In summary, the findings of this study indicated an association between dexmedetomidine and decreased mortality in sepsis patients. Considering the limitations, more high-quality trials are needed to improve the methodology and corroborate the study findings. Further studies are required to determine the population that would benefit the most from this drug and its optimal dosing regimen and infusion duration.

## Conclusions

Optimizing treatment for sepsis patients and improving their outcomes is a worldwide research goal. The findings of this study are valuable for clinical work on sepsis patients. The meta-analysis showed that dexmedetomidine sedation in sepsis patients could decrease mortality compared with benzodiazepines but not with propofol. In addition, dexmedetomidine can significantly decrease inflammatory cytokine levels in sepsis patients compared with other sedatives. Dexmedetomidine might lead to an increased incidence in arrythmias, but its safety profile did not show an increased incidence of total adverse events. Future clinical RCTs are needed to verify the efficacy of dexmedetomidine on the length of the hospital stay and mechanical ventilation time and to determine the sepsis patient population that would benefit the most from this treatment and its optimal dosing regimen.

## Supplementary Information

**Additional file 1**. PRISMA checklist. Sample Search Strategy: **Appendix S1 to Appendix S5**.e-Table S1: mortality timeline.** Appendix S6**. Summary of Findings Table: e-Table S2 to e-Table S8. **Appendix S6.** Summary of Findings Table: e-Table 2 to e-Table 8. e-Table S9: Levels of IL-6 and TNF-α changes at 24 h. e-Table S10: Levels of Alanine Transaminase and Creatinine changes at 24 he-Figure 1: Summarizes the RoB for each individual trial. e-Figure 2: e-Figure_2 Publish bias assessments. e-Figure 3: Effect of dexmedetomidine on mortality. e-Figure 4: Sensitivity analysis based on mortality . e-Figure 5: Effect of dexmedetomidine on ICU stays. e-Figure 6: Forest plot of duration of mechanical ventilation. e-Figure 7: Forest plot of ventilator-free days. e-Figure S8: Effect of dexmedetomidine on levels of IL-6. e-Figure 9: Effect of dexmedetomidine on levels of TNF-α. e-Figure 10: Effect of dexmedetomidine on levels of ALT and Cr. e-Figure 11: Forest plot of Incidence of delirium. e-Figure 12: Forest plot of Incidence of adverse events.

## Data Availability

All data associated with this manuscript are included in the main text and supplementary materials.

## Abbreviations

CI: Confidence interval
TSA: Trial Sequential Analysis
RCT: Randomized-controlled trial
ALT: Alanine transaminase
Cr: Creatine
RoB: Risk of bias
RR: Relative risk
OR: Odds ratio
MD: Mean difference
SMD: Standardized mean difference
IQR: Interquartile ranges
SD: Standard deviation
