# Supplementary material for: Use of dexmedetomidine in patients with sepsis: a systematic review and meta-analysis of randomized-controlled trials
Source: Ann Intensive Care. 2022 Aug 27;12:81. doi: 10.1186/s13613-022-01052-2 (PMC9420168; doi:10.1186/s13613-022-01052-2)
Supplement: Supplementary file 1 — Additional file 1. PRISMA checklist. Sample Search Strategy: Appendix S1 to Appendix S5.e-Table S1: mortality timeline. Appendix S6. Summary of Findings Table: e-Table S2 to e-Table S8. Appendix S6. Summary of Findings Table: e-Table 2 to e-Table 8. e-Table S9: Levels of IL-6 and TNF-α changes at 24 h. e-Table S10: Levels of Alanine Transaminase and Creatinine changes at 24 he-Figure 1: Summarizes the RoB for each individual trial. e-Figure 2: e-Figure_2 Publish bias assessments. e-Figure 3: Effect of dexmedetomidine on mortality. e-Figure 4: Sensitivity analysis based on mortality . e-Figure 5: Effect of dexmedetomidine on ICU stays. e-Figure 6: Forest plot of duration of mechanical ventilation. e-Figure 7: Forest plot of ventilator-free days. e-Figure S8: Effect of dexmedetomidine on levels of IL-6. e-Figure 9: Effect of dexmedetomidine on levels of TNF-α. e-Figure 10: Effect of dexmedetomidine on levels of ALT and Cr. e-Figure 11: Forest plot of Incidence of delirium. e-Figure 12: Forest plot of Incidence of adverse events. [file 13613_2022_1052_MOESM1_ESM.docx]

**Supplementary Online Content**

**PRISMA checklist**

**Sample Search Strategy: Appendix 1 to Appendix 5.**

**e-Table1: mortality timeline**

**Appendix 6. Summary of Findings Table: e-Table 2 to e-Table 8.**

**e-Table 9:** **Levels of IL-6 and TNF-α changes at 24 h**

**e-Table 10: Levels of Alanine Transaminase and Creatinine changes at 24 h**

**e-Figure 1: Summarizes the ROB for each individual trial.**

**e-Figure 2: e-Figure_2 Publish bias assessments.**

**e-Figure 3: Effect of dexmedetomidine on mortality.**

**e-Figure 4: Sensitivity analysis based on mortality .**

**e-Figure 5: Effect of dexmedetomidine on ICU stays.**

**e-Figure 6: Forest plot of duration of mechanical ventilation.**

**e-Figure 7: Forest plot of ventilator-free days.**

**e-Figure 8: Effect of dexmedetomidine on levels of IL-6.**

**e-Figure 9:** **Effect of dexmedetomidine on levels of TNF-α.**

**e-Figure 10: Effect of dexmedetomidine on levels of ALT and Cr.**

**e-Figure 11:** **Forest plot of Incidence of delirium.**

**e-Figure 12: Forest plot of Incidence of adverse events.**

| **Section/topic** | **#** | **Checklist item** | **Reported on page #** |
| --- | --- | --- | --- |
| **TITLE** | | |  |
| Title | 1 | Use of dexmedetomidine in patients with sepsis: a systematic review and meta-analysis of a randomized controlled trial. | 1 |
| **ABSTRACT** | | |  |
| Structured summary | 2 | Provide a structured summary including, as applicable: background; objectives; data sources; study eligibility criteria, participants, and interventions; study appraisal and synthesis methods; results; limitations; conclusions and implications of key findings; systematic review registration number. | 3 |
| **INTRODUCTION** | | |  |
| Rationale | 3 | Describe the rationale for the review in the context of what is already known. | 5 |
| Objectives | 4 | Provide an explicit statement of questions being addressed with reference to participants, interventions, comparisons, outcomes, and study design (PICOS). | 5 |
| **METHODS** | | |  |
| Protocol and registration | 5 | Indicate if a review protocol exists, if and where it can be accessed (e.g., Web address), and, if available, provide registration information including registration number. | 6 |
| Eligibility criteria | 6 | Specify study characteristics (e.g., PICOS, length of follow-up) and report characteristics (e.g., years considered, language, publication status) used as criteria for eligibility, giving rationale. | 6 |
| Information sources | 7 | Describe all information sources (e.g., databases with dates of coverage, contact with study authors to identify additional studies) in the search and date last searched. | 6 |
| Search | 8 | Present full electronic search strategy for at least one database, including any limits used, such that it could be repeated. | 6 |
| Study selection | 9 | State the process for selecting studies (i.e., screening, eligibility, included in systematic review, and, if applicable, included in the meta-analysis). | 6-7 |
| Data collection process | 10 | Describe method of data extraction from reports (e.g., piloted forms, independently, in duplicate) and any processes for obtaining and confirming data from investigators. | 7 |
| Data items | 11 | List and define all variables for which data were sought (e.g., PICOS, funding sources) and any assumptions and simplifications made. | 7 |
| Risk of bias in individual studies | 12 | Describe methods used for assessing risk of bias of individual studies (including specification of whether this was done at the study or outcome level), and how this information is to be used in any data synthesis. | 7-8 |
| Summary measures | 13 | State the principal summary measures (e.g., risk ratio, difference in means). | 8 |
| Synthesis of results | 14 | Describe the methods of handling data and combining results of studies, if done, including measures of consistency (e.g., I^2^) for each meta-analysis. | 8-9 |

Page 1 of 2

| **Section/topic** | **#** | **Checklist item** | **Reported on page #** |
| --- | --- | --- | --- |
| Risk of bias across studies | 15 | Specify any assessment of risk of bias that may affect the cumulative evidence (e.g., publication bias, selective reporting within studies). | 9 |
| Additional analyses | 16 | Describe methods of additional analyses (e.g., sensitivity or subgroup analyses, meta-regression), if done, indicating which were pre-specified. | 9 |
| **RESULTS** | | |  |
| Study selection | 17 | Give numbers of studies screened, assessed for eligibility, and included in the review, with reasons for exclusions at each stage, ideally with a flow diagram. | 9-10 |
| Study characteristics | 18 | For each study, present characteristics for which data were extracted (e.g., study size, PICOS, follow-up period) and provide the citations. | 10 |
| Risk of bias within studies | 19 | Present data on risk of bias of each study and, if available, any outcome level assessment (see item 12). | 10 |
| Results of individual studies | 20 | For all outcomes considered (benefits or harms), present, for each study: (a) simple summary data for each intervention group (b) effect estimates and confidence intervals, ideally with a forest plot. | 10, 19 |
| Synthesis of results | 21 | Present results of each meta-analysis done, including confidence intervals and measures of consistency. | 11-12 |
| Risk of bias across studies | 22 | Present results of any assessment of risk of bias across studies (see Item 15). | 10, 23-25 |
| Additional analysis | 23 | Give results of additional analyses, if done (e.g., sensitivity or subgroup analyses, meta-regression [see Item 16]). | 12 |
| **DISCUSSION** | | |  |
| Summary of evidence | 24 | Summarize the main findings including the strength of evidence for each main outcome; consider their relevance to key groups (e.g., healthcare providers, users, and policy makers). | 13-14 |
| Limitations | 25 | Discuss limitations at study and outcome level (e.g., risk of bias), and at review-level (e.g., incomplete retrieval of identified research, reporting bias). | 16 |
| Conclusions | 26 | Provide a general interpretation of the results in the context of other evidence, and implications for future research. | 14-15 |
| **FUNDING** | | |  |
| Funding | 27 | Describe sources of funding for the systematic review and other support (e.g., supply of data); role of funders for the systematic review. | 17 |

*From:*  Moher D, Liberati A, Tetzlaff J, Altman DG, The PRISMA Group (2009). Preferred Reporting Items for Systematic Reviews and Meta-Analyses: The PRISMA Statement. PLoS Med 6(6): e1000097. doi:10.1371/journal.pmed1000097

For more information, visit: **www.prisma-statement.org**.

Page 2 of 2

**Appendix 1. EMBASE, PUBMED, Web of Science Search Strategy**

EMBASE:

No. Query Results

#8. #5 AND #6 AND #7 54

#7. 'randomized controlled trial':ab,ti OR 1,017,160

'randomized':ab,ti OR 'placebo':ab,ti

#6. #3 OR #4 14,445

#5. #1 OR #2 322,756

#4. 'mpv-1440':ab,ti OR 'mpv 1440':ab,ti OR 190

'mpv1440':ab,ti OR 'precedex':ab,ti OR

'dexmedetomidine hydrochloride':ab,ti OR

'hydrochloride, dexmedetomidine':ab,ti

#3. 'dexmedetomidine'/exp 14,440

#2. 'bloodstream infection':ab,ti OR 'bloodstream 48,711

infections':ab,ti OR 'infection,

bloodstream':ab,ti OR 'pyemia':ab,ti OR

'pyemias':ab,ti OR 'pyohemia':ab,ti OR

'pyohemias':ab,ti OR 'pyaemia':ab,ti OR

'pyaemias':ab,ti OR 'septicemia':ab,ti OR

'septicemias':ab,ti OR 'poisoning, blood':ab,ti

OR 'blood poisoning':ab,ti OR 'blood

poisonings':ab,ti OR 'poisonings, blood':ab,ti OR

'severe sepsis':ab,ti OR 'sepsis, severe':ab,ti

#1. 'sepsis'/exp 305,703

PUBMED:

8 ((("Sepsis"[Mesh]) OR (((((((((((((((((Bloodstream Infection[Title/Abstract]) OR (Bloodstream Infections[Title/Abstract])) OR (Infection, Bloodstream[Title/Abstract])) OR (Pyemia[Title/Abstract])) OR (Pyemias[Title/Abstract])) OR (Pyohemia[Title/Abstract])) OR (Pyohemias[Title/Abstract])) OR (Pyaemia[Title/Abstract])) OR (Pyaemias[Title/Abstract])) OR (Septicemia[Title/Abstract])) OR (Septicemias[Title/Abstract])) OR (Poisoning, Blood[Title/Abstract])) OR (Blood Poisoning[Title/Abstract])) OR (Blood Poisonings[Title/Abstract])) OR (Poisonings, Blood[Title/Abstract])) OR (Severe Sepsis[Title/Abstract])) OR (Sepsis, Severe[Title/Abstract]))) AND (("Dexmedetomidine"[Mesh]) OR ((((((MPV-1440[Title/Abstract]) OR (MPV 1440[Title/Abstract])) OR (MPV1440[Title/Abstract])) OR (Precedex[Title/Abstract])) OR (Dexmedetomidine Hydrochloride[Title/Abstract])) OR (Hydrochloride, Dexmedetomidine[Title/Abstract])))) AND (randomized controlled trial[Publication Type] OR randomized[Title/Abstract] OR placebo[Title/Abstract]) ("Sepsis"[MeSH Terms] OR ("bloodstream infection"[Title/Abstract] OR "bloodstream infections"[Title/Abstract] OR "infection bloodstream"[Title/Abstract] OR "Pyemia"[Title/Abstract] OR "Pyemias"[Title/Abstract] OR "Pyohemia"[Title/Abstract] OR "Pyohemias"[Title/Abstract] OR "Pyaemia"[Title/Abstract] OR "Septicemia"[Title/Abstract] OR "Septicemias"[Title/Abstract] OR "poisoning blood"[Title/Abstract] OR "blood poisoning"[Title/Abstract] OR "blood poisonings"[Title/Abstract] OR (("poisoned"[All Fields] OR "Poisoning"[MeSH Terms] OR "Poisoning"[All Fields] OR "Poisonings"[All Fields] OR "Poisoning"[MeSH Subheading] OR "poisonous"[All Fields] OR "poisons"[Pharmacological Action] OR "poisons"[MeSH Terms] OR "poisons"[All Fields] OR "poison"[All Fields]) AND "Blood"[Title/Abstract]) OR "severe sepsis"[Title/Abstract] OR "sepsis severe"[Title/Abstract])) AND ("Dexmedetomidine"[MeSH Terms] OR ("MPV-1440"[Title/Abstract] OR "MPV-1440"[Title/Abstract] OR "MPV1440"[Title/Abstract] OR "Precedex"[Title/Abstract] OR "dexmedetomidine hydrochloride"[Title/Abstract] OR "hydrochloride dexmedetomidine"[Title/Abstract])) AND ("randomized controlled trial"[Publication Type] OR "randomized"[Title/Abstract] OR "placebo"[Title/Abstract]) 21 10:39:22

7 randomized controlled trial[Publication Type] OR randomized[Title/Abstract] OR placebo[Title/Abstract] "randomized controlled trial"[Publication Type] OR "randomized"[Title/Abstract] OR "placebo"[Title/Abstract] 929,767 10:36:06

6 ("Dexmedetomidine"[Mesh]) OR ((((((MPV-1440[Title/Abstract]) OR (MPV 1440[Title/Abstract])) OR (MPV1440[Title/Abstract])) OR (Precedex[Title/Abstract])) OR (Dexmedetomidine Hydrochloride[Title/Abstract])) OR (Hydrochloride, Dexmedetomidine[Title/Abstract])) "Dexmedetomidine"[MeSH Terms] OR "MPV-1440"[Title/Abstract] OR "MPV-1440"[Title/Abstract] OR "MPV1440"[Title/Abstract] OR "Precedex"[Title/Abstract] OR "dexmedetomidine hydrochloride"[Title/Abstract] OR "hydrochloride dexmedetomidine"[Title/Abstract] 4,575 10:35:45

5 (((((MPV-1440[Title/Abstract]) OR (MPV 1440[Title/Abstract])) OR (MPV1440[Title/Abstract])) OR (Precedex[Title/Abstract])) OR (Dexmedetomidine Hydrochloride[Title/Abstract])) OR (Hydrochloride, Dexmedetomidine[Title/Abstract]) "MPV-1440"[Title/Abstract] OR "MPV-1440"[Title/Abstract] OR "MPV1440"[Title/Abstract] OR "Precedex"[Title/Abstract] OR "dexmedetomidine hydrochloride"[Title/Abstract] OR "hydrochloride dexmedetomidine"[Title/Abstract] 120 10:35:38

4 "Dexmedetomidine"[Mesh] Most Recent "Dexmedetomidine"[MeSH Terms] 4,534 10:34:33

3 ("Sepsis"[Mesh]) OR (((((((((((((((((Bloodstream Infection[Title/Abstract]) OR (Bloodstream Infections[Title/Abstract])) OR (Infection, Bloodstream[Title/Abstract])) OR (Pyemia[Title/Abstract])) OR (Pyemias[Title/Abstract])) OR (Pyohemia[Title/Abstract])) OR (Pyohemias[Title/Abstract])) OR (Pyaemia[Title/Abstract])) OR (Pyaemias[Title/Abstract])) OR (Septicemia[Title/Abstract])) OR (Septicemias[Title/Abstract])) OR (Poisoning, Blood[Title/Abstract])) OR (Blood Poisoning[Title/Abstract])) OR (Blood Poisonings[Title/Abstract])) OR (Poisonings, Blood[Title/Abstract])) OR (Severe Sepsis[Title/Abstract])) OR (Sepsis, Severe[Title/Abstract])) "Sepsis"[MeSH Terms] OR ("bloodstream infection"[Title/Abstract] OR "bloodstream infections"[Title/Abstract] OR "infection bloodstream"[Title/Abstract] OR "Pyemia"[Title/Abstract] OR "Pyemias"[Title/Abstract] OR "Pyohemia"[Title/Abstract] OR "Pyohemias"[Title/Abstract] OR "Pyaemia"[Title/Abstract] OR "Septicemia"[Title/Abstract] OR "Septicemias"[Title/Abstract] OR "poisoning blood"[Title/Abstract] OR "blood poisoning"[Title/Abstract] OR "blood poisonings"[Title/Abstract] OR (("poisoned"[All Fields] OR "Poisoning"[MeSH Terms] OR "Poisoning"[All Fields] OR "Poisonings"[All Fields] OR "Poisoning"[MeSH Subheading] OR "poisonous"[All Fields] OR "poisons"[Pharmacological Action] OR "poisons"[MeSH Terms] OR "poisons"[All Fields] OR "poison"[All Fields]) AND "Blood"[Title/Abstract]) OR "severe sepsis"[Title/Abstract] OR "sepsis severe"[Title/Abstract]) 176,549 10:33:11

2 ((((((((((((((((Bloodstream Infection[Title/Abstract]) OR (Bloodstream Infections[Title/Abstract])) OR (Infection, Bloodstream[Title/Abstract])) OR (Pyemia[Title/Abstract])) OR (Pyemias[Title/Abstract])) OR (Pyohemia[Title/Abstract])) OR (Pyohemias[Title/Abstract])) OR (Pyaemia[Title/Abstract])) OR (Pyaemias[Title/Abstract])) OR (Septicemia[Title/Abstract])) OR (Septicemias[Title/Abstract])) OR (Poisoning, Blood[Title/Abstract])) OR (Blood Poisoning[Title/Abstract])) OR (Blood Poisonings[Title/Abstract])) OR (Poisonings, Blood[Title/Abstract])) OR (Severe Sepsis[Title/Abstract])) OR (Sepsis, Severe[Title/Abstract]) "bloodstream infection"[Title/Abstract] OR "bloodstream infections"[Title/Abstract] OR "infection bloodstream"[Title/Abstract] OR "Pyemia"[Title/Abstract] OR "Pyemias"[Title/Abstract] OR "Pyohemia"[Title/Abstract] OR "Pyohemias"[Title/Abstract] OR "Pyaemia"[Title/Abstract] OR "Septicemia"[Title/Abstract] OR "Septicemias"[Title/Abstract] OR "poisoning blood"[Title/Abstract] OR "blood poisoning"[Title/Abstract] OR "blood poisonings"[Title/Abstract] OR (("poisoned"[All Fields] OR "Poisoning"[MeSH Terms] OR "Poisoning"[All Fields] OR "Poisonings"[All Fields] OR "Poisoning"[MeSH Subheading] OR "poisonous"[All Fields] OR "poisons"[Pharmacological Action] OR "poisons"[MeSH Terms] OR "poisons"[All Fields] OR "poison"[All Fields]) AND "Blood"[Title/Abstract]) OR "severe sepsis"[Title/Abstract] OR "sepsis severe"[Title/Abstract] 64,320 10:32:50

1 "Sepsis"[Mesh] Most Recent "Sepsis"[MeSH Terms] 132,943 10:29:39

Web of Science:

4

#3 AND #2 AND #1 56

3

TS=（randomized controlled trial OR randomized OR placebo OR RCT ） 1,142,012

2

TS=（Dexmedetomidine OR MPV-1440 OR MPV 1440 OR MPV1440 OR Precedex OR Dexmedetomidine Hydrochloride OR Hydrochloride, Dexmedetomidine） 8,992

1

TS=（sepsis OR Bloodstream Infection OR Bloodstream Infections OR Infection, Bloodstream OR Pyemia OR Pyemias OR Pyohemia OR Pyohemias OR Pyaemia OR Pyaemias OR Septicemia OR Septicemias OR Poisoning, Blood OR Blood Poisoning OR Blood Poisonings OR Poisonings, Blood OR Severe Sepsis OR Sepsis, Severe） 311,390

**Appendix 2. COCHRANE CENTRAL Search Strategy**

COCHRANE Controlled Clinical Trials Registry:

#1 sepsis 13138

#2 (Bloodstream Infection):ab,ti,kw OR (Bloodstream Infections):ab,ti,kw OR (Infection, Bloodstream):ab,ti,kw OR (Pyemia):ab,ti,kw OR (Pyemias):ab,ti,kw OR (Pyohemia):ab,ti,kw OR (Pyohemias):ab,ti,kw OR (Pyaemia):ab,ti,kw OR (Pyaemias):ab,ti,kw OR (Septicemia):ab,ti,kw OR (Septicemias):ab,ti,kw OR (Poisoning, Blood):ab,ti,kw OR (Blood Poisoning):ab,ti,kw OR (Blood Poisonings):ab,ti,kw OR (Poisonings, Blood):ab,ti,kw OR (Severe Sepsis):ab,ti,kw OR (Sepsis, Severe):ab,ti,kw 5851

#3 #1 OR #2 15547

#4 Dexmedetomidine 6138

#5 (MPV-1440):ab,ti,kw OR (MPV 1440):ab,ti,kw OR (MPV1440):ab,ti,kw OR (Precedex):ab,ti,kw OR (Dexmedetomidine Hydrochloride):ab,ti,kw OR (Hydrochloride, Dexmedetomidine):ab,ti,kw 181

#6 #4 OR #5 6143

#7 (randomized controlled trial):ab,ti,kw OR (randomized):ab,ti,kw OR (placebo):ab,ti,kw OR (RCT):ab,ti,kw 1107982

#8 #3 AND #6 AND #7 47

**Appendix 3. PROSPERO Search Strategy**

PROSPERO:

dexmedetomidine AND sepsis

15 studies found

**Appendix 4. GOOGLE Scholar Search Strategy**

Google Scholar:

dexmedetomidine AND sepsis AND (RCT or randomized controlled trial or randomized or placebo)

First five pages of results (50 citations) included)

**Appendix 5. CLINICALTRIALS.gov Search Strategy**

Clinical Trials.gov

Advanced search, no date limit applied

Condition or disease: (sepsis)

AND

Other terms: (dexmedetomidine)

20 studies

**e-Table1 mortality timeline**

| **Study author and year** | **No. of patients DEX/control** | **Timeline** |
| --- | --- | --- |
| Cai et al., 2019 | 30/30 | 28-day mortality:3/4 |
| Cioccari et al., 2020 | 44/39 | ICU mortality: 6/10;Hospital mortality: 9/12;90-day mortality: 12/13 |
| Hughes et al., 2021 | 214/208 | 90-day mortality: 81/82 |
| Kawazoe et al., 2017 | 100/101 | 28-day mortality: 19/28 |
| Lei et al., 2016 | 29/29 | 28-day mortality:3/4 |
| Liu et al., 2020 | 100/100 | 28-day mortality: 64/62 |
| Memiş  et al., 2009 | 20/20 | overall ICU mortality: 3/4 |
| Meng et al., 2014 | 20/20 | 28-day mortality: 1/2 |
| Pandharipande et al., 2010 | 31/32 | 28-day mortality: 5/13 |
| Qian et al., 2017 | 60/60 | 28-day mortality: 7/18 |
| Ren et al., 2017 | 25/25 | 28-day mortality: 6/8 |
| Sigler et al., 2018 | 17/19 | 28-day mortality:9/8 |
| Tasdogan et al., 2009 | 20/20 | 28-day mortality:3/5 |
| Wang et al., 2019 | 31/32 | 28-day mortality: 2/10 |
| Wei et al., 2020 | 60/59 | 30-day mortality:8/11 |
| Zhang et al., 2020 | 25/25 | 28-day mortality: 4/2 |
| Zheng et al., 2019 | 32/30 | 7-day mortality:4/12 |
| Zhou et al., 2017 | 40/40 | 28-day mortality:2/3 |

**Appendix 6 : Summary of Findings Table**

**e-Table 2：**

**Question:** incidence of total adverse events compared to other sedatives for Patients with sepsis

| **Certainty assessment** | | | | | | | **№ of patients** | | **Effect** | | **Certainty** | **Importance** |
| --- | --- | --- | --- | --- | --- | --- | --- | --- | --- | --- | --- | --- |
| **№ of studies** | **Study design** | **Risk of bias** | **Inconsistency** | **Indirectness** | **Imprecision** | **Other considerations** | **Incidence of adverse** | **placebo** | **Relative (95% CI)** | **Absolute (95% CI)** |  |  |
| **Incidence of Total Adverse Events** | | | | | | | | | | | | |
| 6 | randomised trials | not serious | not serious | not serious | serious^a^ | none | 67/293 (22.9%) | 53/288 (18.4%) | **RR 1.27** (0.69 to 2.36) | **50 more per 1,000** (from 57 fewer to 250 more) | ⨁⨁⨁◯ Moderate | CRITICAL |
| **Arrhythmia** | | | | | | | | | | | | |
| 4 | randomised trials | not serious | not serious | not serious | not serious | none | 23/202 (11.4%) | 8/198 (4.0%) | **RR 2.69** (1.19 to 6.08) | **68 more per 1,000** (from 8 more to 205 more) | ⨁⨁⨁⨁ High | CRITICAL |
| **Hypotension** | | | | | | | | | | | | |
| 3 | randomised trials | serious | not serious | not serious | serious^a^ | none | 11/134 (8.2%) | 10/128 (7.8%) | **RR 1.04** (0.46 to 2.36) | **3 more per 1,000** (from 42 fewer to 106 more) | ⨁⨁◯◯ Low | IMPORTANT |

**CI:** confidence interval; **OR:** odds ratio; **RR:** risk ratio

**Explanations**

a. Wide confidence intervals do not exclude important benefit or harm which lowers our certainty in effect.

**e-Table 3:**

**Question:** inflammatory cytokine compared to other sedatives for Patients with sepsis

| **Certainty assessment** | | | | | | | **№ of patients** | | **Effect** | | **Certainty** | **Importance** |
| --- | --- | --- | --- | --- | --- | --- | --- | --- | --- | --- | --- | --- |
| **№ of studies** | **Study design** | **Risk of bias** | **Inconsistency** | **Indirectness** | **Imprecision** | **Other considerations** | **inflammatory** | **placebo** | **Relative (95% CI)** | **Absolute (95% CI)** |  |  |
| **Baseline levels of IL-6** | | | | | | | | | | | | |
| 4 | randomised trials | serious^a^ | serious^b^ | not serious | very serious^c^ | all plausible residual confounding would reduce the demonstrated effect | 176 | 176 | - | SMD **0.31 lower** (0.54 lower to 0.08 lower) | ⨁◯◯◯ Very low | IMPORTANT |
| **Baseline levels of TNF-α** | | | | | | | | | | | | |
| 4 | randomised trials | serious^a^ | not serious | not serious | not serious | all plausible residual confounding would reduce the demonstrated effect | 176 | 176 | - | SMD **0.16 lower** (0.37 lower to 0.05 higher) | ⨁⨁⨁⨁ High | IMPORTANT |
| **Levels of IL-6 at 24 h** | | | | | | | | | | | | |
| 4 | randomised trials | serious^a^ | not serious | not serious | serious^d^ | none | 176 | 176 | - | SMD **2.15 lower** (3.25 lower to 1.05 lower) | ⨁⨁◯◯ Low | IMPORTANT |
| **Levels of TNF-α at 24 h** | | | | | | | | | | | | |
| 3 | randomised trials | serious^a^ | not serious | not serious | not serious^d^ | none | 176 | 176 | - | SMD **1.07 lower** (1.92 lower to 0.22 lower) | ⨁⨁⨁◯ Moderate | IMPORTANT |

**CI:** confidence interval; **SMD:** standardised mean difference

**Explanations**

a. 3 studies have high ROB

b. High I2 (36%) and non-overlapping confidence intervals suggest important inconsistency which lowers our certainty in effect

c. There is significant heterogeneity among studies.

d. Wide confidence intervals that do not exclude serious benefit or harm

**e-Table 4:**

**Question:** ICU stays compared to other sedatives for Patients with sepsis

| **Certainty assessment** | | | | | | | **№ of patients** | | **Effect** | | **Certainty** | **Importance** |
| --- | --- | --- | --- | --- | --- | --- | --- | --- | --- | --- | --- | --- |
| **№ of studies** | **Study design** | **Risk of bias** | **Inconsistency** | **Indirectness** | **Imprecision** | **Other considerations** | **ICUstay** | **placebo** | **Relative (95% CI)** | **Absolute (95% CI)** |  |  |
| **ICU Stays** | | | | | | | | | | | | |
| 9 | randomised trials | not serious | not serious | not serious | not serious | none | 330 | 329 | - | MD **0.22 lower** (0.85 lower to 0.41 higher) | ⨁⨁⨁⨁ High | CRITICAL |

**CI:** confidence interval; **MD:** mean difference

**e-Table 5:**

**Question:** use of mechanical ventilation compared to other sedatives for Patients with sepsis

| **Certainty assessment** | | | | | | | **№ of patients** | | **Effect** | | **Certainty** | **Importance** |
| --- | --- | --- | --- | --- | --- | --- | --- | --- | --- | --- | --- | --- |
| **№ of studies** | **Study design** | **Risk of bias** | **Inconsistency** | **Indirectness** | **Imprecision** | **Other considerations** | **ventilation** | **placebo** | **Relative (95% CI)** | **Absolute (95% CI)** |  |  |
| **Duration of Mechanical Ventilation** | | | | | | | | | | | | |
| 6 | randomised trials | not serious | not serious | not serious | not serious | all plausible residual confounding would reduce the demonstrated effect | 231 | 229 | - | MD **0.12 lower** (1.1 lower to 1.35 higher) | ⨁⨁⨁⨁ High | CRITICAL |
| **Ventilator-free days** | | | | | | | | | | | | |
| 2 | randomised trials | not serious | very serious^a^ | not serious | serious^b^ | none | 314 | 309 | - | MD **0.29 higher** (1.81 lower to 2.39 higher) | ⨁◯◯◯ Very low | IMPORTANT |

**CI:** confidence interval; **MD:** mean difference

**Explanations**

a. High I2 (63%) and non-overlapping confidence intervals suggest important inconsistency which lowers our certainty in effect

b. Low number of events below optimal information size contributing to imprecision which lowers our certainty in effect

**e-Table 6:**

**Question:** delirium compared to other sedatives for Patients with sepsis

| **Certainty assessment** | | | | | | | **№ of patients** | | **Effect** | | **Certainty** | **Importance** |
| --- | --- | --- | --- | --- | --- | --- | --- | --- | --- | --- | --- | --- |
| **№ of studies** | **Study design** | **Risk of bias** | **Inconsistency** | **Indirectness** | **Imprecision** | **Other considerations** | **delirium** | **placebo** | **Relative (95% CI)** | **Absolute (95% CI)** |  |  |
| **Incidence of delirium** | | | | | | | | | | | | |
| 2 | randomised trials | serious^a^ | not serious | not serious | serious^b^ | none | 45/131 (34.4%) | 47/133 (35.3%) | **RR 0.98** (0.72 to 1.33) | **7 fewer per 1,000** (from 99 fewer to 117 more) | ⨁⨁◯◯ Low | IMPORTANT |

**CI:** confidence interval; **RR:** risk ratio

**Explanations**

a. one study has high ROB

b. Low number of events below optimal information size contributing to imprecision which lowers our certainty in effect

**e-Table 7:**

**Question:** levels of creatinine compared to other sedatives for Patients with sepsis

| **Certainty assessment** | | | | | | | **№ of patients** | | **Effect** | | **Certainty** | **Importance** |
| --- | --- | --- | --- | --- | --- | --- | --- | --- | --- | --- | --- | --- |
| **№ of studies** | **Study design** | **Risk of bias** | **Inconsistency** | **Indirectness** | **Imprecision** | **Other considerations** | **肌酐** | **placebo** | **Relative (95% CI)** | **Absolute (95% CI)** |  |  |
| **Baseline levels of creatinine** | | | | | | | | | | | | |
| 3 | randomised trials | serious^a^ | not serious | not serious | serious^b^ | none | 110 | 109 | - | SMD **0.06 higher** (0.2 lower to 0.33 higher) | ⨁⨁◯◯ Low | IMPORTANT |
| **Levels of creatinine at 24 h** | | | | | | | | | | | | |
| 3 | randomised trials | serious^a^ | serious^c^ | not serious | serious^b^ | none | 110 | 109 | - | SMD **0.31 lower** (0.9 lower to 0.27 higher) | ⨁◯◯◯ Very low | IMPORTANT |

**CI:** confidence interval; **SMD:** standardised mean difference

**Explanations**

a. Two studies had some concerns

b. Low number of events below optimal information size contributing to imprecision which lowers our certainty in effect

c. High I2 (76%) and non-overlapping confidence intervals suggest important inconsistency which lowers our certainty in effect

**e-Table 8:**

**Question:** ALT compared to other sedatives for Patients with sepsis

| **Certainty assessment** | | | | | | | **№ of patients** | | **Effect** | | **Certainty** | **Importance** |
| --- | --- | --- | --- | --- | --- | --- | --- | --- | --- | --- | --- | --- |
| **№ of studies** | **Study design** | **Risk of bias** | **Inconsistency** | **Indirectness** | **Imprecision** | **Other considerations** | **ALT** | **placebo** | **Relative (95% CI)** | **Absolute (95% CI)** |  |  |
| **Baseline levels of ALT** | | | | | | | | | | | | |
| 3 | randomised trials | serious^a^ | not serious | not serious | serious^b^ | none | 110 | 109 | - | SMD **0.14 lower** (0.46 lower to 0.17 higher) | ⨁⨁◯◯ Low | IMPORTANT |
| **Levels of ALT at 24 h** | | | | | | | | | | | | |
| 3 | randomised trials | serious^a^ | not serious | not serious | serious^b^ | none | 110 | 109 | - | SMD **0.24 lower** (0.58 lower to 0.1 higher) | ⨁⨁◯◯ Low | IMPORTANT |

**CI:** confidence interval; **SMD:** standardised mean difference; **ALT:**alanine transaminase

**Explanations**

a. Two studies had some concerns

b. Low number of events below optimal information size contributing to imprecision which lowers our certainty in effect

**e-Table 9:** Levels of IL-6 and TNF-α changes at 24 h

|  |  | | Dexmedetomidine | | | | Control | | | |
| --- | --- | --- | --- | --- | --- | --- | --- | --- | --- | --- |
|  |  | | Sample size | IL-6 |  | TNF-α | Sample size |  | IL-6 | TNF-α |
| Tasdogan,2009 | |  | 20 | 388.6 ± 209.8， after 24h 253.1 ± 103.6 |  | 11.72 ± 6.02，after 24h 14.66 ± 4.40 | 20 |  | 460.1 ± 227.3，after 24h 511.3 ± 374.8 | 13.97 ± 6.29，after 24h 21.21 ± 11.37 |
| Zhu,2017 | |  | 47 | 48.4±7.2；after 24h 88±14.2 |  | 16.4±2.6； after 24h 33.5±3 | 47 |  | 46.8±8.3；after 24h 121.4±12.2 | 16.5±6.2；after 24h 39.8±2.5 |
| Chen,2018 | |  | 80 | 516.45±43.16；after 24h 429.81±37.89 |  | 230.43±30.20 after 24h 199.39±28.29 | 80 |  | 540.19±49.18；after 24h 449.34±48.94 | 230.19±31.09，after 24h 211.43±20.97 |
| Zhang,2020 | |  | 25 | 475. 32±26. 43，after 24h 340. 71±10. 37 |  | 20. 59±3. 16； after 24h 14. 95±3. 37 | 25 |  | 475. 58±26. 65； after 24h 390. 89±12. 26 | 20. 64±3. 08； after 24h 18. 83±4. 46 |
| Zhang,2013 | |  | 50 | 14.79±5.2ng/L，after 24h 69.34±18.1 |  | 0.71±0.18μg/ L after 24h 0.74±0.18 | 50 |  | 17.86±8.77 after 24h 96.56±42.57 | 0.78±0.23 after 24h 0.93±0.29 |
| Wu,2018 | |  | 48 | 134.59±28.32,after 24h 153.65±31.54 |  | 161.18±23.84，after 24h 191.35±29.07 | 48 |  | 140.26±22.57，after 24h 192.07±27.39 | 169.23±21.26，after 24h 256.82±26.33 |
| Wang,2016 | |  | 28 | 476.96±30.246,after 24h 341.39±33.355 |  | 21.12 ± 1.846，after 24h 15.18 ± 2.091 | 28 |  | 475.11 ± 55.473，after 24h 391.21 ± 58.294 | 21.48 ± 2.276，after 24h 18.29 ± 5.518 |

**e-Table 10:** Levels of Alanine Transaminase and Creatinine changes at 24 h

|  |  | | Dexmedetomidine | | | | Control | | | |
| --- | --- | --- | --- | --- | --- | --- | --- | --- | --- | --- |
|  |  | | Sample size | Cr |  | ALT | Sample size |  | Cr | ALT |
| Memiş,2009 | |  | 20 | 40.4±9；after 24h 39.5±5.7 |  | 140.4±9；after 24h39.5 ± 5.7 | 20 |  | 44.4±2.5；after 24h 39±7.8 |  |
| Wei et, 2020 | |  | 60 | 87. 53±16.98；after 24h 59.65±10.96 |  | 87. 53 ± 16. 98；after 24h59. 65 ± 10. 96 | 59 |  | 88.65±17.76；after 24h 65.37±12.42 |  |
| Cai, 2019 | |  | 30 | 83.6±50.31；after 24h 79.83±43.33 |  | 65.23±53.43 after 24h 56.83±48.6 | 30 |  | 83.5±50.31；after 24h 80.7±42.59 | 65.47±40.55，after 24h 58.5±39.17 |

**
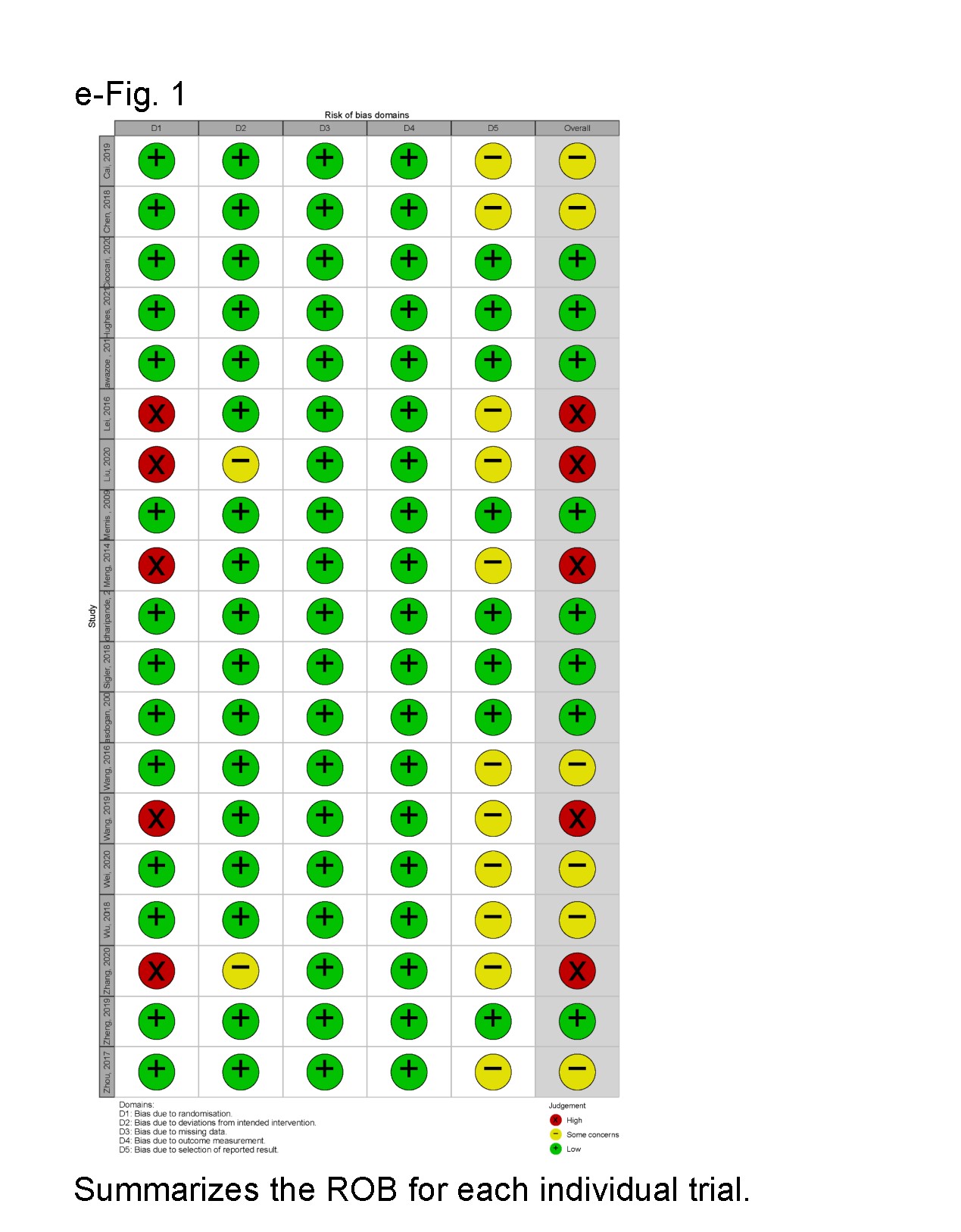
**


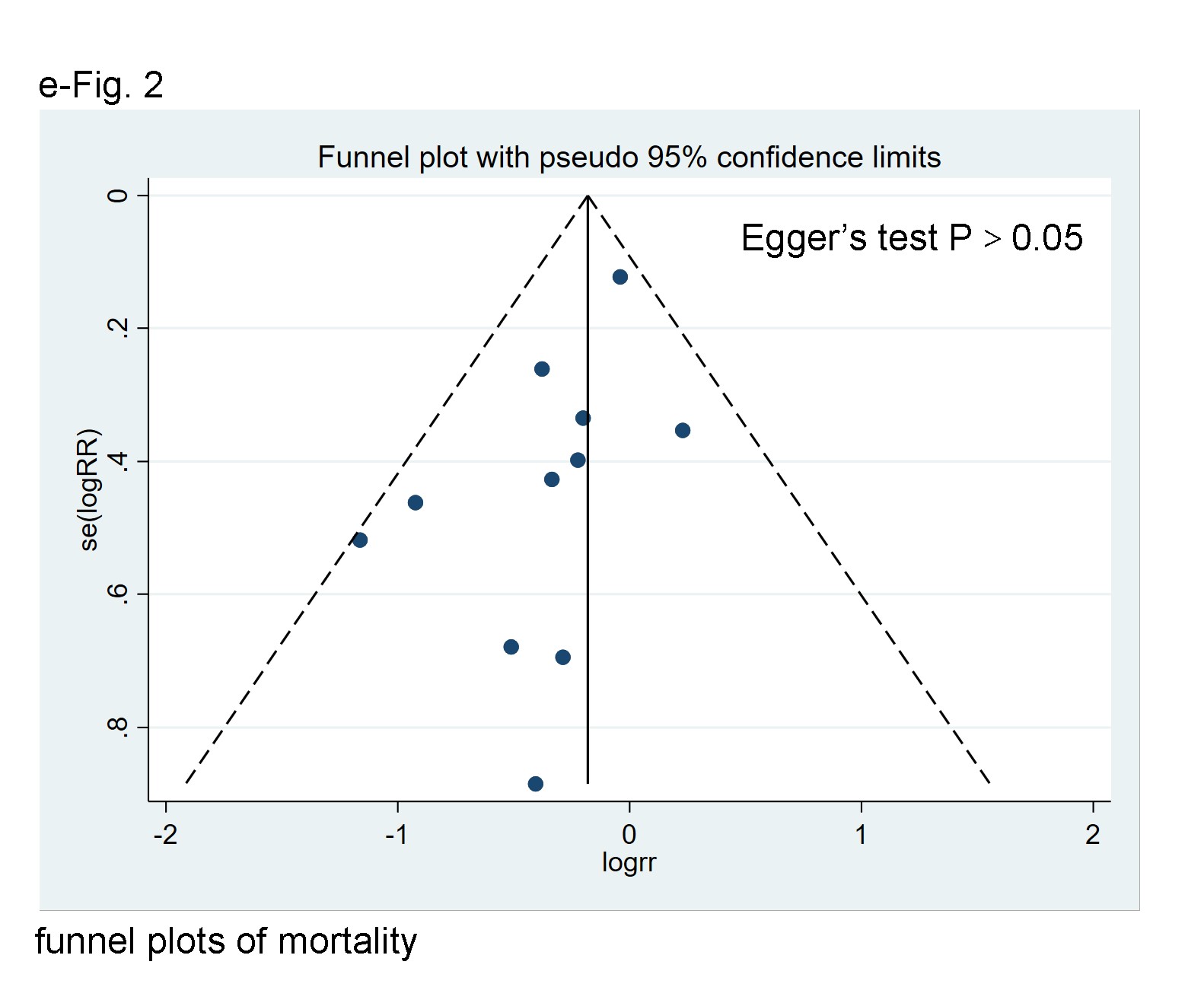


**
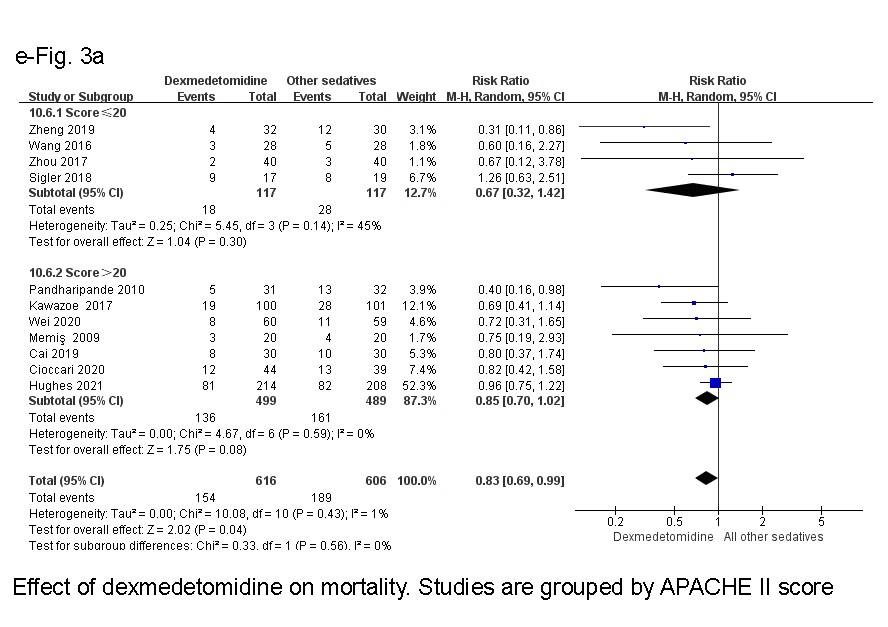
**
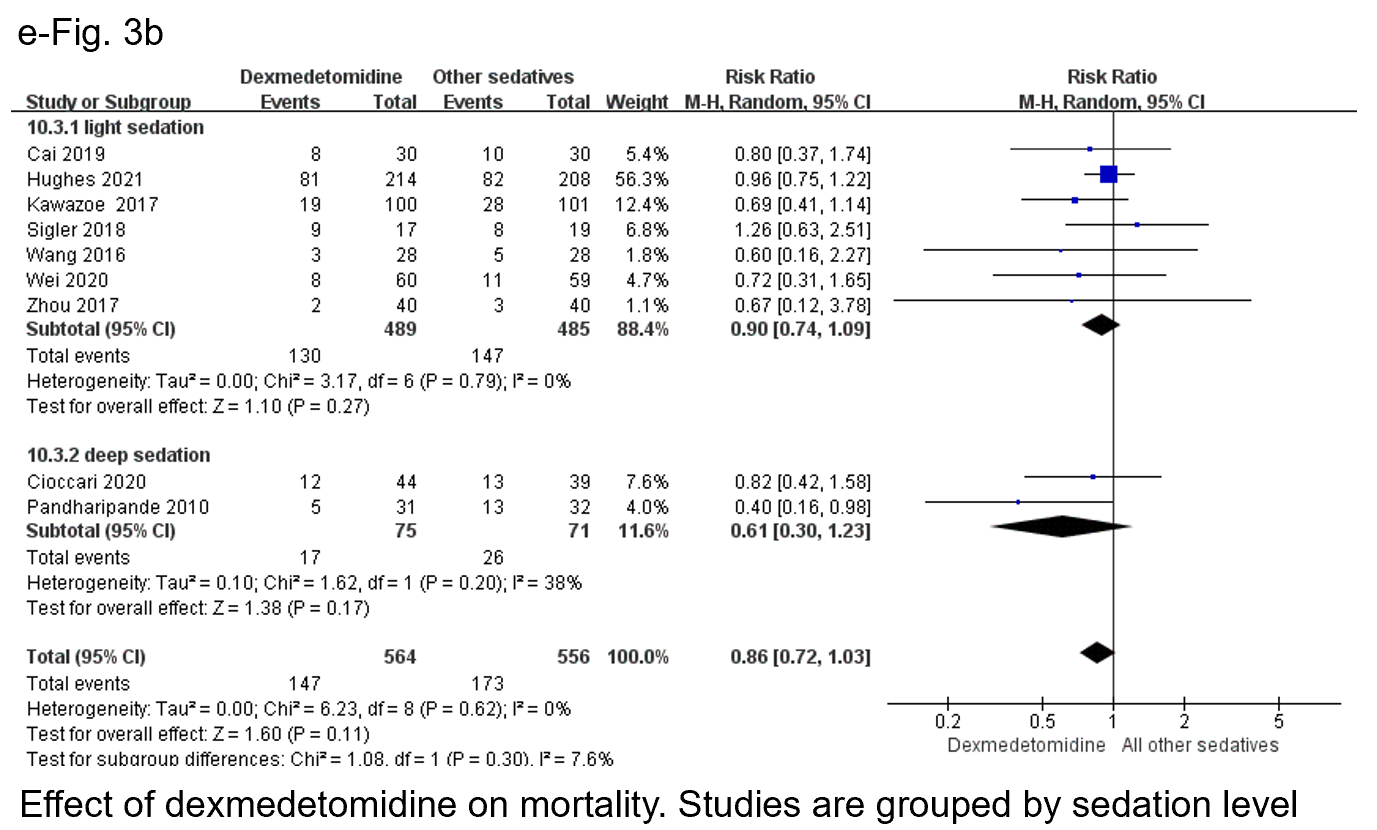
**
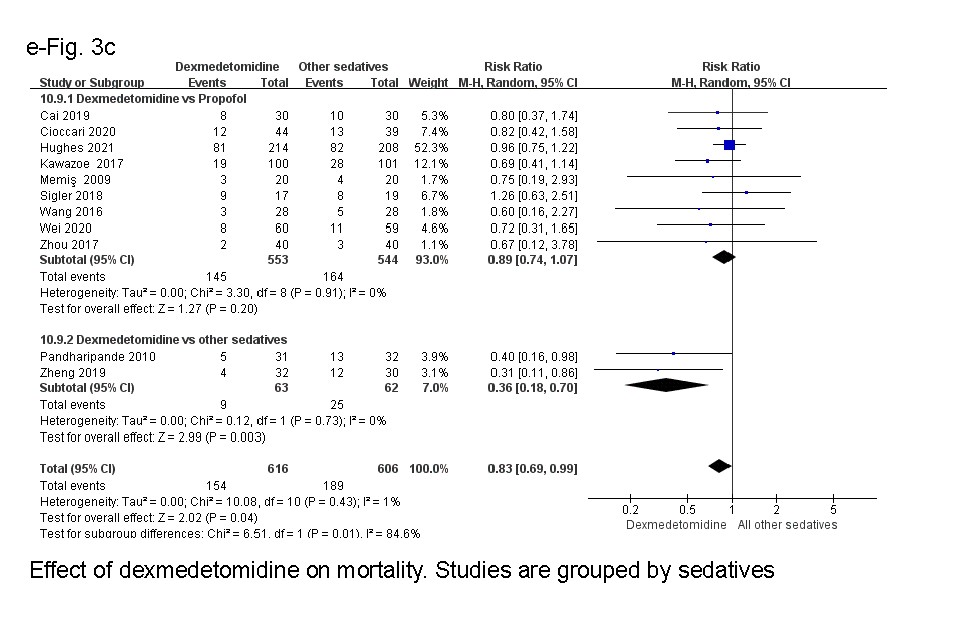
**

**
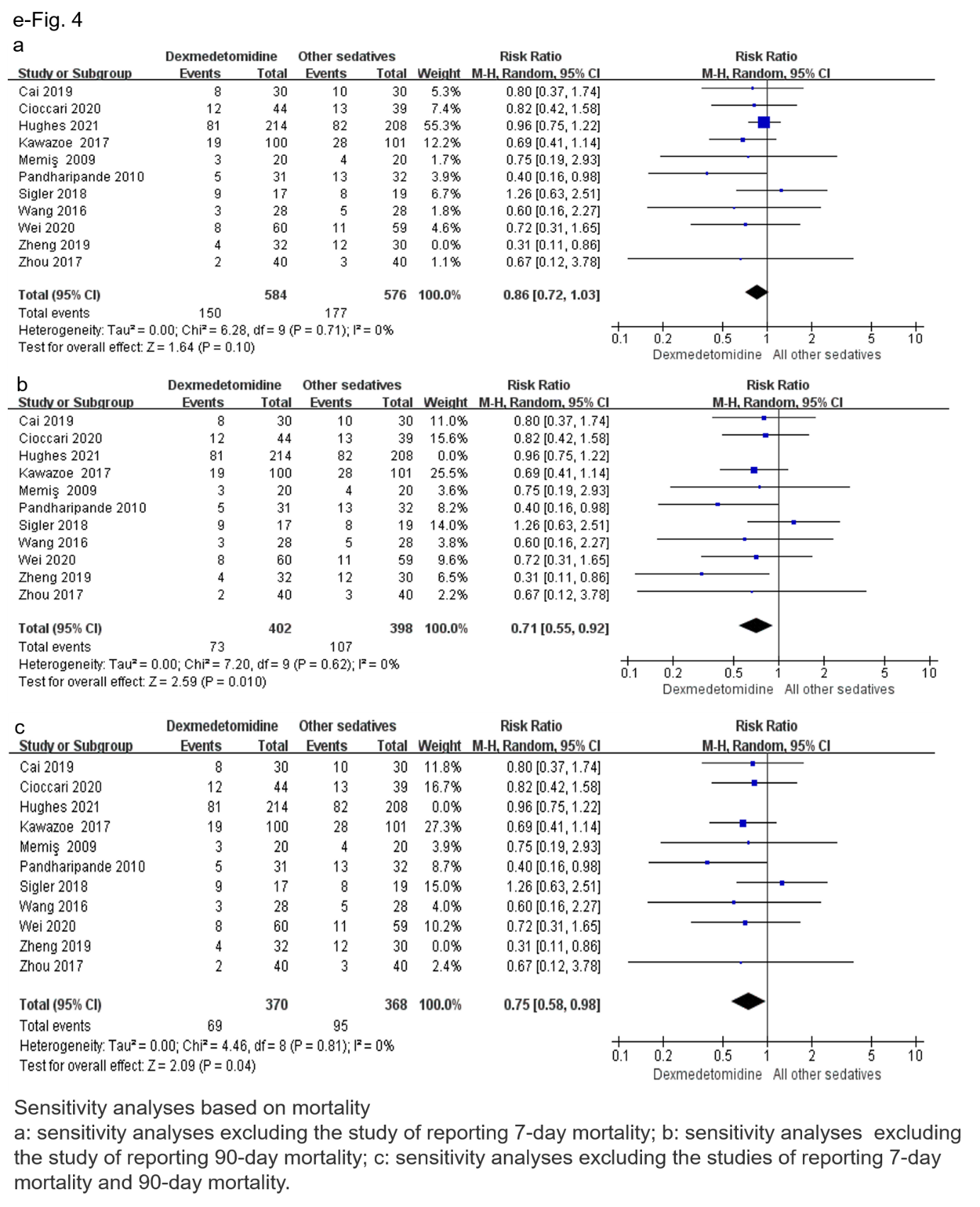
**

**
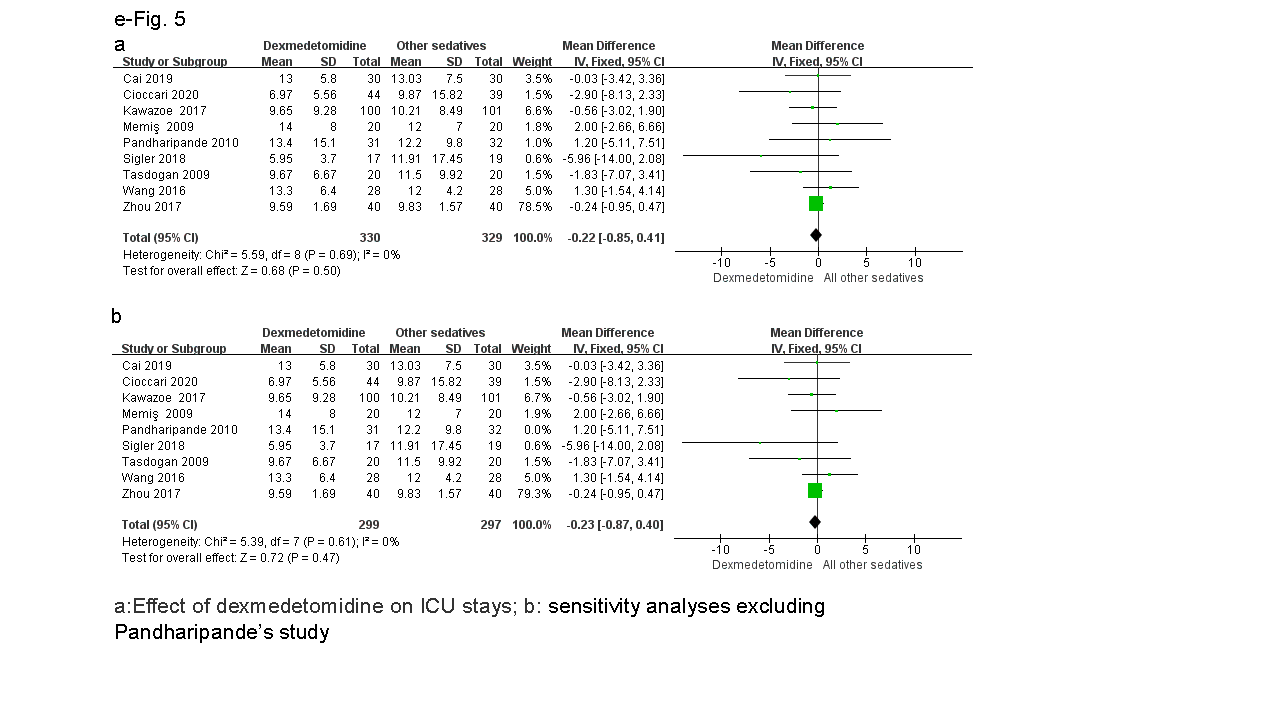
**

**
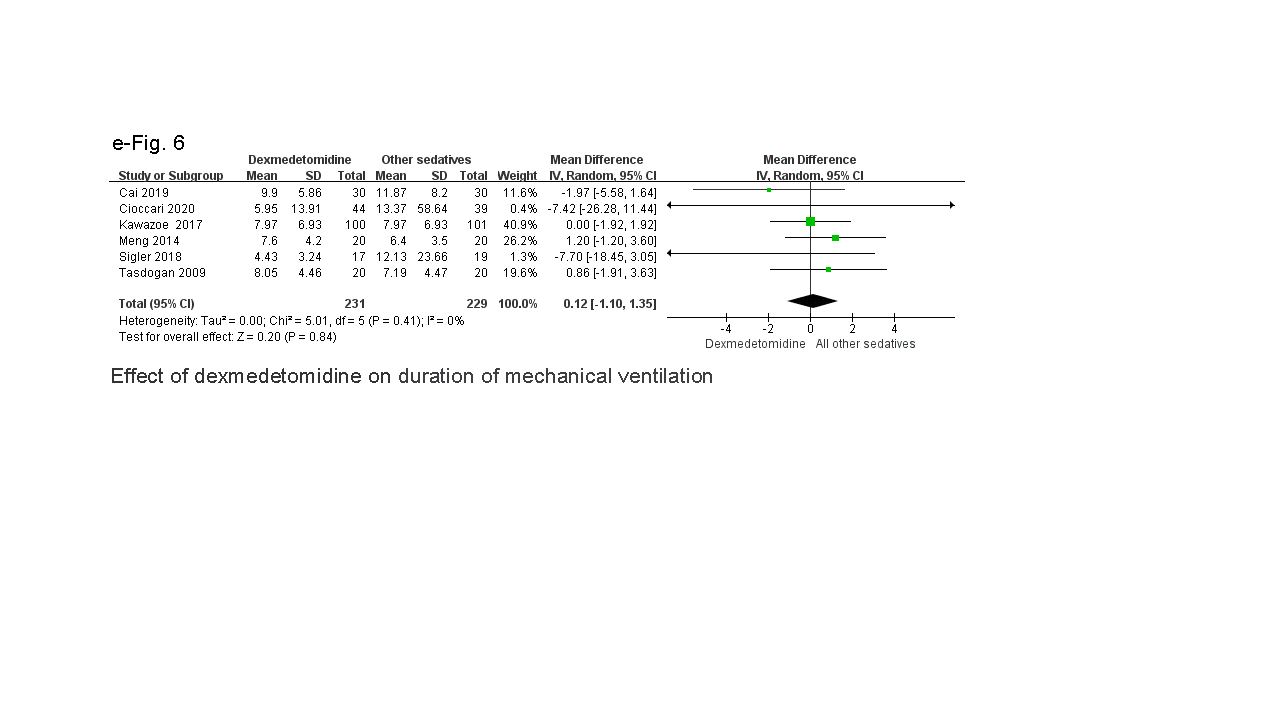
**

**
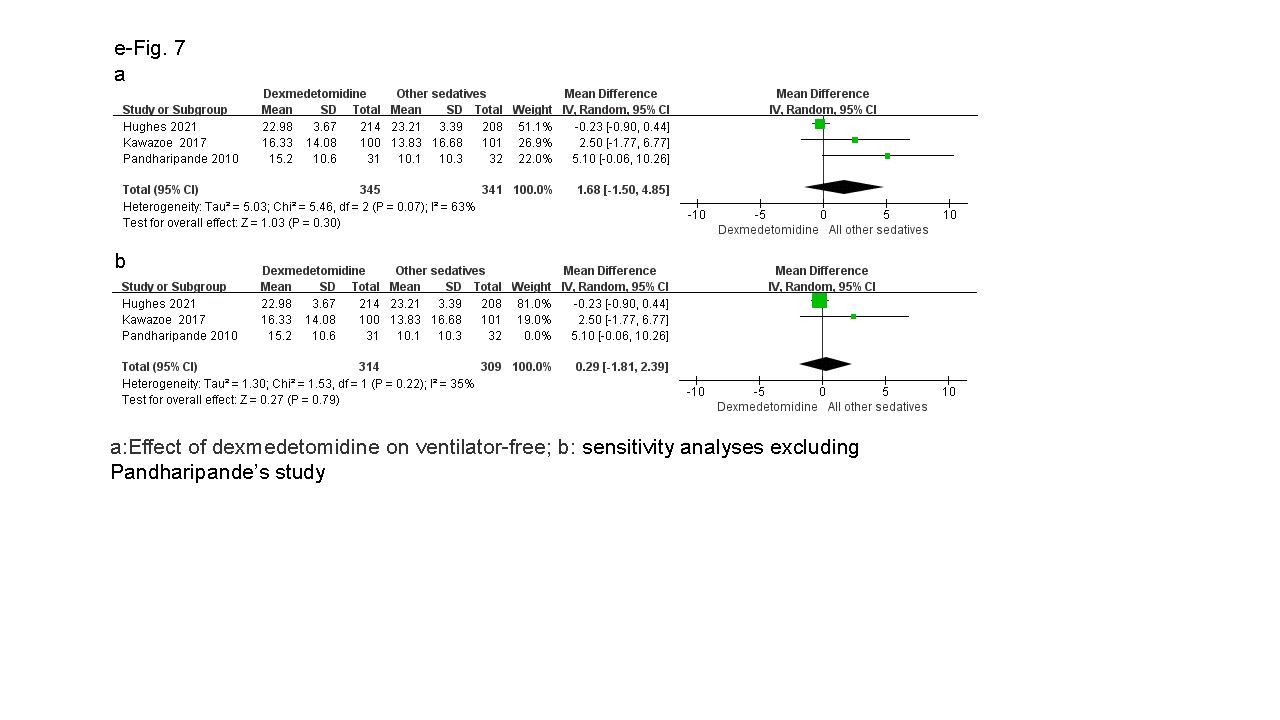
**

**
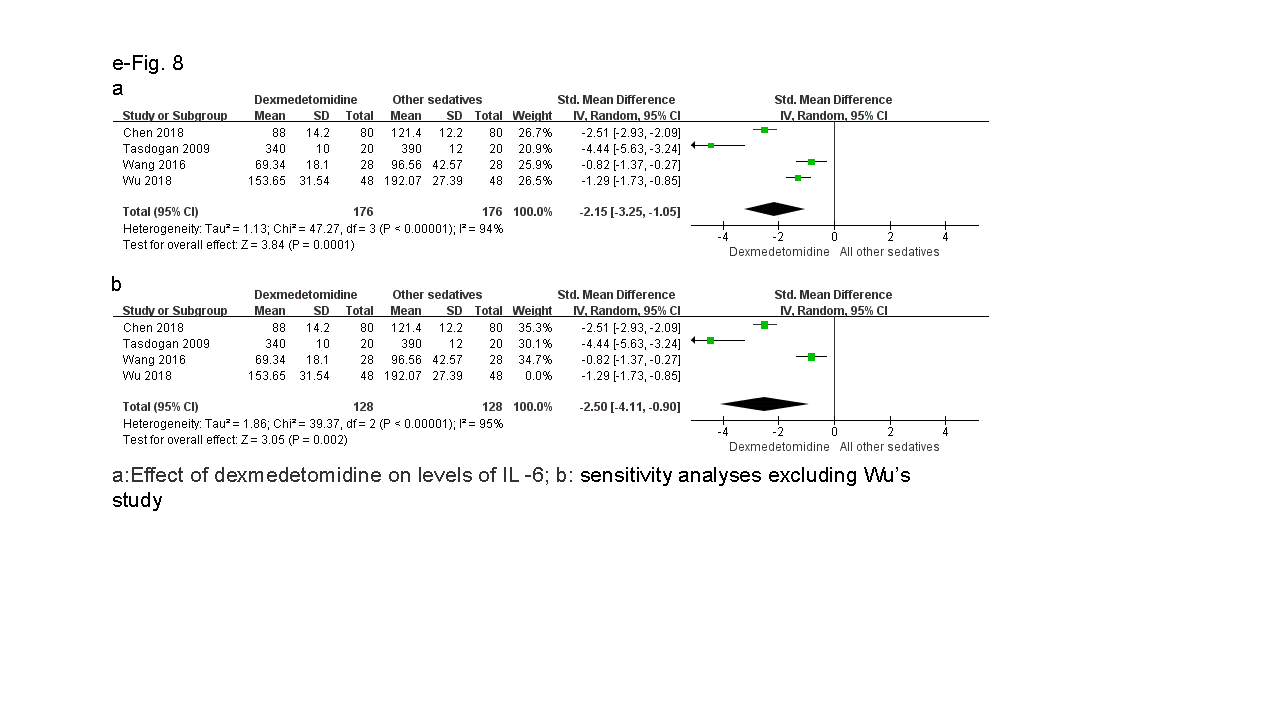
**

**
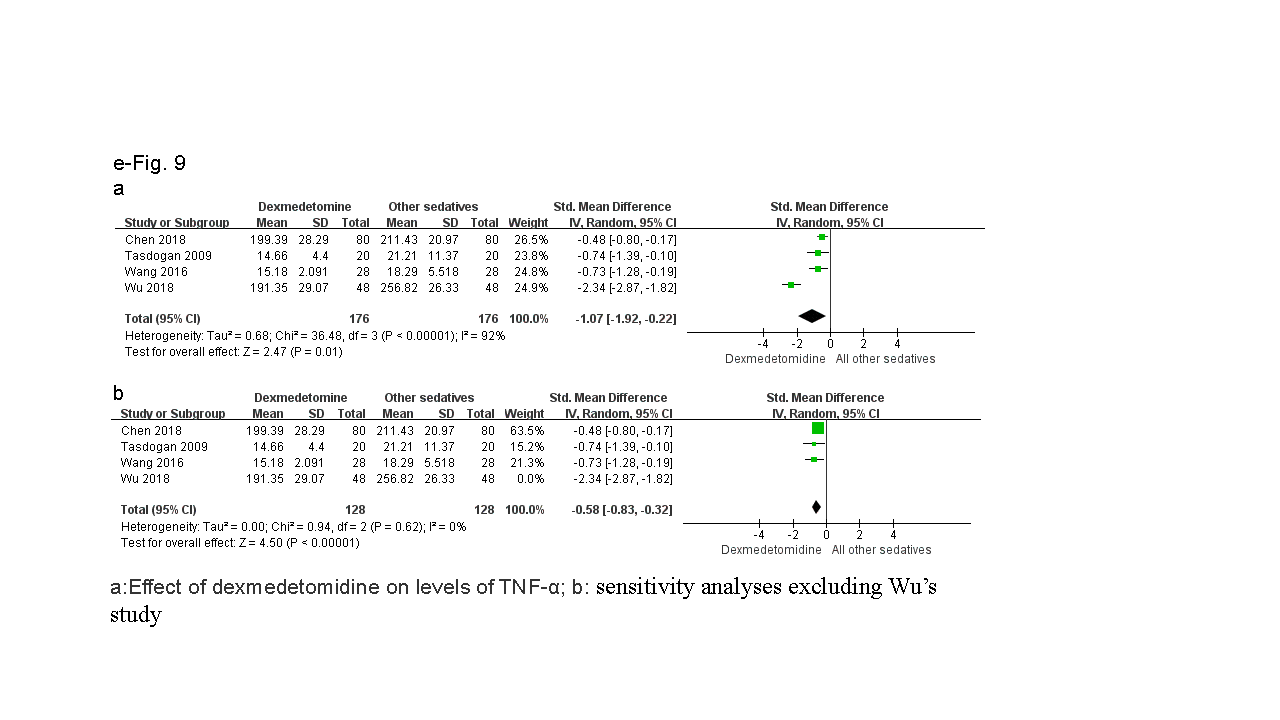
**


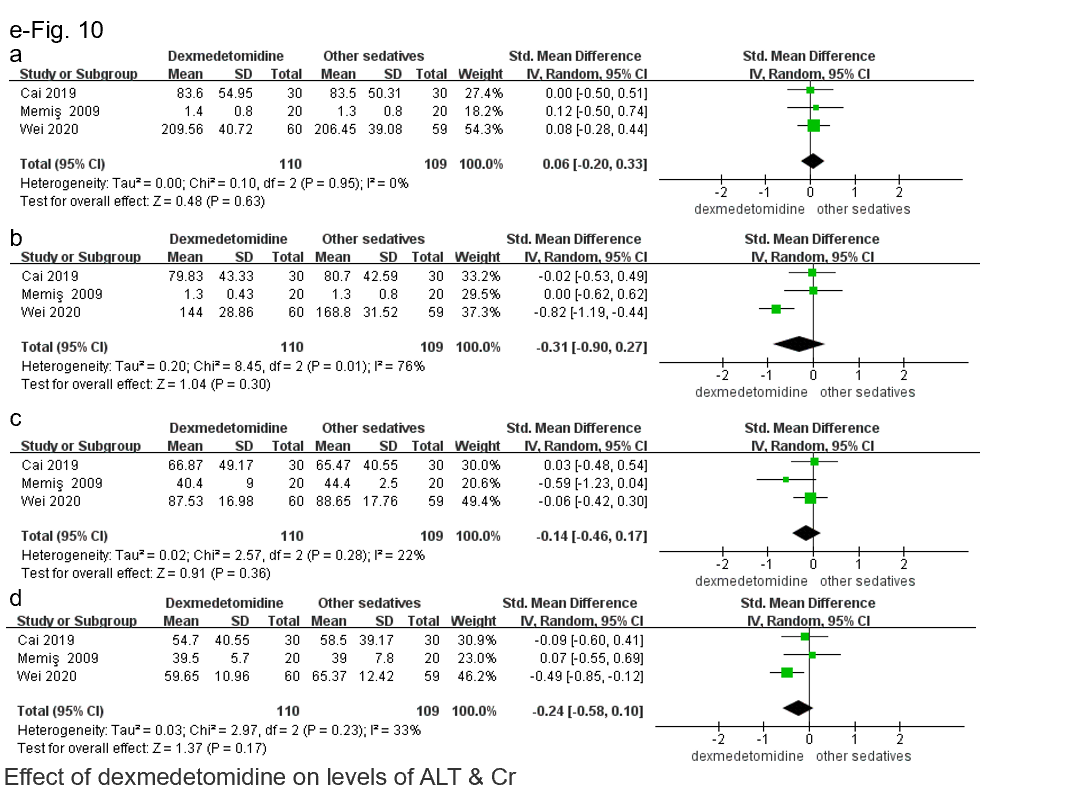


a: Levels of Cr before sedatives; b: Levels of Cr after 24 hours with sedatives;

c: Levels of ALT before sedatives; d: Levels of ALT after 24 hours with sedatives.

**
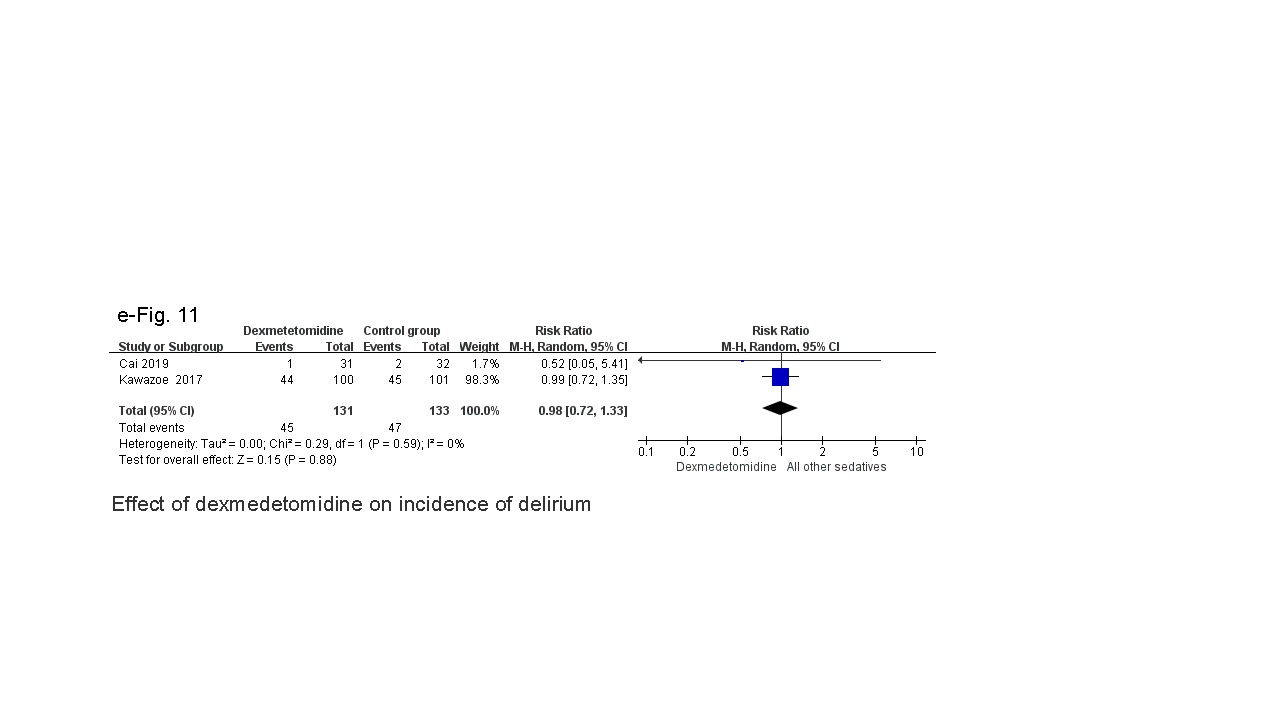
**


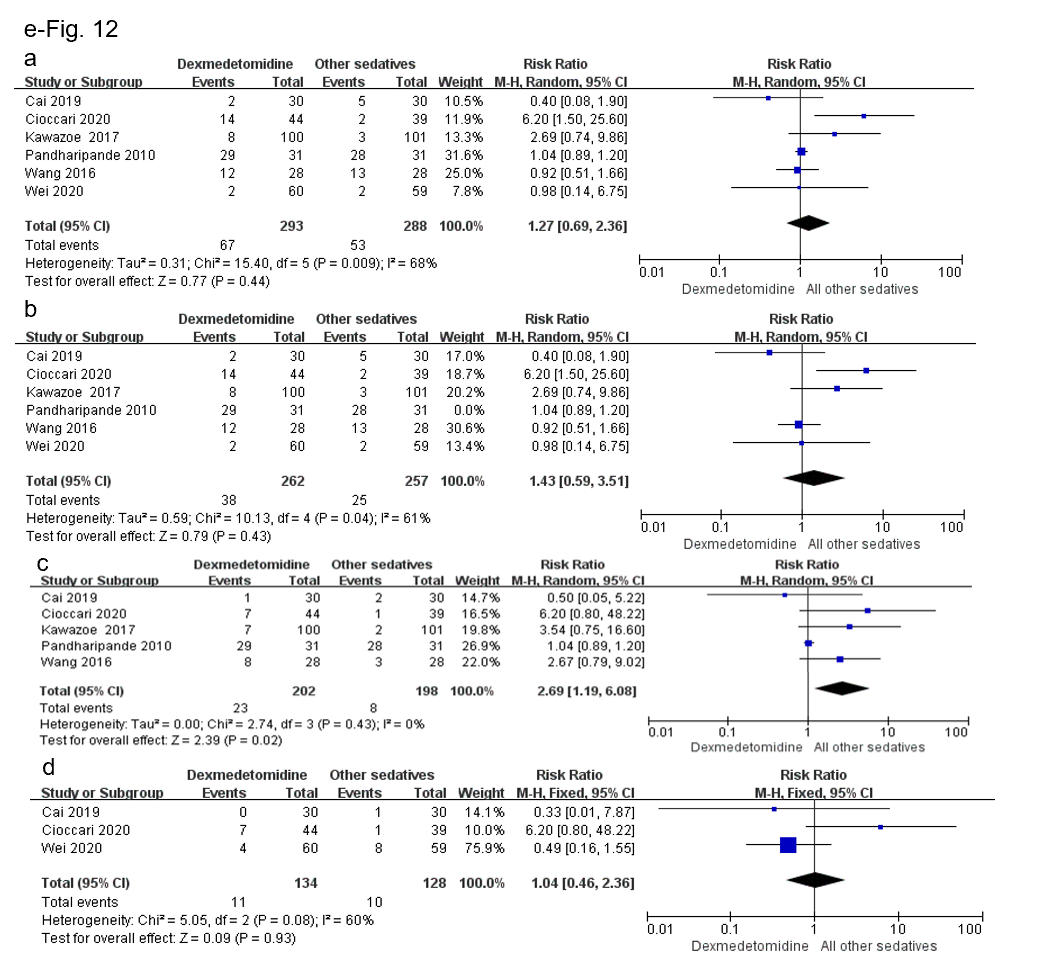


a:Effect of dexmedetomidine on incidence of adverse events; b: sensitivity analyses excluding Pandharipande’s study; c: effect of dexmedetomidine on incidence of arrhythmia; d: effect of dexmedetomidine on incidence of hypotension.
